# Organ-specific metabolic pathways distinguish prediabetes, type 2 diabetes, and normal tissues

**DOI:** 10.1016/j.xcrm.2022.100763

**Published:** 2022-10-04

**Authors:** Klev Diamanti, Marco Cavalli, Maria J. Pereira, Gang Pan, Casimiro Castillejo-López, Chanchal Kumar, Filip Mundt, Jan Komorowski, Atul S. Deshmukh, Matthias Mann, Olle Korsgren, Jan W. Eriksson, Claes Wadelius

**Affiliations:** 1 Department of Immunology, Genetics and Pathology, Science for Life Laboratory, Uppsala University, Uppsala, Sweden; 2 Department of Medical Sciences, Clinical Diabetes and Metabolism, Uppsala University, Uppsala, Sweden; 3 Translational Science & Experimental Medicine, Early Cardiovascular, Renal and Metabolism, BioPharmaceuticals R&D, AstraZeneca, Gothenburg, Sweden; 4 Karolinska Institutet/AstraZeneca Integrated CardioMetabolic Center (KI/AZ ICMC), Department of Medicine, Novum, Huddinge, Sweden; 5 Novo Nordisk Foundation Center for Protein Research, Faculty of Health Sciences, University of Copenhagen, Copenhagen, Denmark; 6 Department of Oncology-Pathology, Karolinska Institute, Stockholm, Sweden; 7 Science for Life Laboratory, Department of Cell and Molecular Biology, Uppsala University, Uppsala, Sweden; 8 Institute of Computer Science, Polish Academy of Sciences, Warsaw, Poland; 9 Washington National Primate Research Center, Seattle, WA, USA; 10 Swedish Collegium for Advanced Study, Uppsala, Sweden; 11 Novo Nordisk Foundation Center for Basic Metabolic Research, Faculty of Health and Medical Science, University of Copenhagen, Copenhagen, Denmark; 12 Department of Proteomics and Signal Transduction, Max Planck Institute of Biochemistry, Martinsried, Germany; 13 Department of Medicine, University of Gothenburg, Gothenburg, Sweden

**Keywords:** type 2 diabetes, T2D, prediabetes, multi-tissue, pancreatic islets, visceral adipose tissue, skeletal muscle, liver, mass spectrometry, proteomics

## Abstract

Environmental and genetic factors cause defects in pancreatic islets driving type 2 diabetes (T2D) together with the progression of multi-tissue insulin resistance. Mass spectrometry proteomics on samples from five key metabolic tissues of a cross-sectional cohort of 43 multi-organ donors provides deep coverage of their proteomes. Enrichment analysis of Gene Ontology terms provides a tissue-specific map of altered biological processes across healthy, prediabetes (PD), and T2D subjects. We find widespread alterations in several relevant biological pathways, including increase in hemostasis in pancreatic islets of PD, increase in the complement cascade in liver and pancreatic islets of PD, and elevation in cholesterol biosynthesis in liver of T2D. Our findings point to inflammatory, immune, and vascular alterations in pancreatic islets in PD that are hypotheses to be tested for potential contributions to hormonal perturbations such as impaired insulin and increased glucagon production. This multi-tissue proteomic map suggests tissue-specific metabolic dysregulations in T2D.

## Highlights

• Mass spectrometry proteomics of 5 key metabolic tissues from 43 multi-organ donors
• The study provides a map of tissue-specific metabolic dysregulations in PD and T2D
• Inflammatory, immune, and vascular processes are altered in pancreatic islets in PD
• Lipid and mitochondrial pathways are dysregulated in liver and VAT/muscle in T2D

## Graphical abstract

**Figure:**
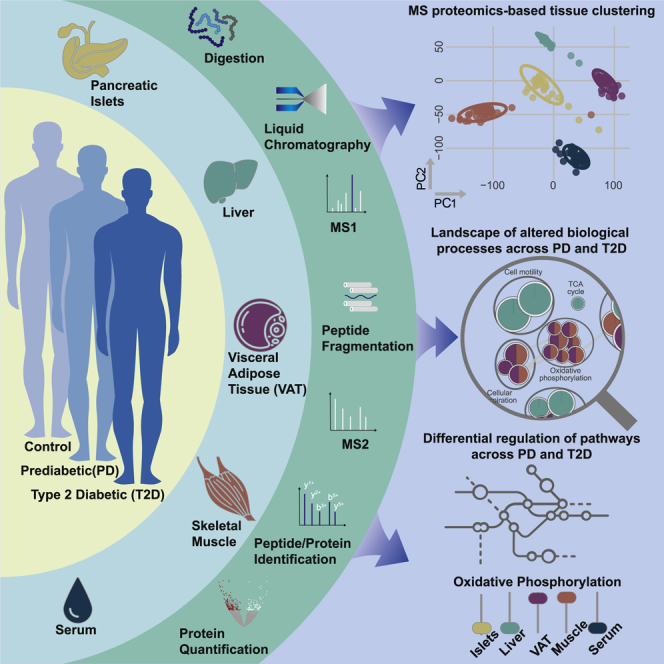

## Teaser

Diamanti et al. map the proteome of five key metabolic tissues of 43 healthy, prediabetes (PD), and type 2 diabetes (T2D) multi-organ donors. The exploration of tissue-specific biological processes indicates that pancreatic islets show multiple biological alterations in PD, while other tissues demonstrate widespread perturbations in T2D.

## Introduction

Insufficient secretion of insulin from pancreatic β cells and poor sensitivity to insulin from multiple tissues are important for the development of type 2 diabetes (T2D) that, in turn, is often followed by late complications, disability, increased mortality, and increased health care costs. High-energy diet, genetic factors, and limited physical activity leading to obesity are predisposing factors that lead to an increased risk for T2D.^1^^,^^2^ Besides genetic factors, various cellular components, such as proteins and metabolites, have been reported to be consistently altered in T2D, partly driven by environmental factors.^3^, ^4^, ^5^ Several studies have associated the effects of insufficient insulin with specific cellular deregulations, including glucose levels and lipid deposition in tissues and fatty acid uptake and metabolism.^6^, ^7^, ^8^

The major tissues for the development of T2D include pancreatic islets, various adipose depots, skeletal muscle, liver, intestine, and the central nervous system, but they remain largely understudied.^9^ Instead, the majority of studies exploring T2D have focused on easily accessible tissues such as biofluids that exhibit cellular and molecular alterations reflecting events that may take place in the other, primary tissues, thus providing indirect evidence.^9^^,^^10^ Exploring alterations in biological pathways across the various metabolically relevant tissues and distinct states of T2D would provide an information-rich holistic view of the primary events leading to the disease.

Liquid chromatography (LC) coupled with mass spectrometry (MS) is a methodology that is routinely employed for the identification and quantification of proteins from tissue samples, thus ultimately aiming at discoveries of biological and medical relevance.^11^ Technological advances, including sample preparation, data acquisition, and computational processing pipelines, have enhanced the overall analytical capacity of MS proteomics.^12^^,^^13^ MS proteomics has been utilized in a spectrum of tissues to study multiple diseases spanning from cerebrospinal fluid for Alzheimer’s disease to brain for medulloblastoma to induced pluripotent stem cells differentiated into myoblasts for T2D.^14^, ^15^, ^16^ A recent review in insulin-target proteomics of various organs highlighted different pathological processes related to insulin resistance (IR). Specifically, increase in immune- and fibrosis-related proteins in adipose tissue, increase in branched-chain amino acids and mitochondrial proteins in liver, and excessive availability of fatty acids in skeletal muscle have been strongly associated with increased IR.^17^

In this cross-sectional study, we used tissue samples from 43 multi-organ donors and MS-based proteomics to build a map of altered biological processes in prediabetes (PD) and T2D in visceral adipose tissue (VAT), liver, skeletal muscle, pancreatic islets, and serum. This resource provides an extensive coverage of the proteome of multiple tissues relevant to T2D. We confirmed a substantial fraction of well-characterized markers from the literature, such as the significant downregulation of the citric acid (TCA) cycle in VAT and skeletal muscle, but more importantly, we identified various biological signals and pathways through comparisons across tissues that substantially expand knowledge on PD and T2D. A network of comparisons among healthy control subjects (CTRLs), subjects with PD, and subjects with T2D provided an overview of responses of tissues and highlighted tissue-specific patterns of altered biological processes. In summary, we provide a resource of protein levels and enriched biological processes in the most important tissues involved in the development of T2D.

## Results

**Figure 1:**
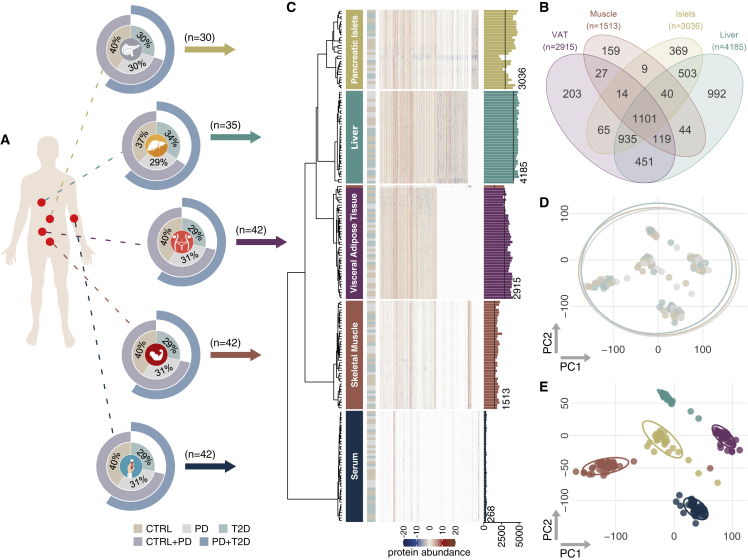
Overview of the study and exploration of the proteomics dataset (A) Schematic overview of tissue samples. Doughnut charts show the distribution of CTRL, PD, and T2D subjects. The two outer circles show the fraction of subjects belonging to the merged groups of CTRL + PD and PD + T2D. (B) Venn diagram that summarizes tissue-shared and tissue-specific proteins. (C) Hierarchical clustering of protein abundancies across samples. Proteins were clustered using the function *ward*.*D* from the R package *stats* based on *1-r*, where *r* is the Pearson correlation coefficient. The dendrogram and the heatmap illustrate clustering and protein intensity, respectively. Tissues are indicated by color and text on the right-hand side of the dendrogram, and the T2D status is shown in the next column. The right-most bar plot shows number of identified proteins in each sample prior to filtering, and the black line marks the number of proteins retained after filtering. (D and E) Principal-component analysis (PCA) of the processed proteomics data of all tissues. The PCA was performed using the function *pca* of the R library *pcaMethods*. (D) Colored by T2D groups; (E) colored by tissues.

A cohort of 43 multi-organ donors of five key metabolic tissues, including VAT, liver, skeletal muscle, pancreatic islets, and serum, was characterized by normoglycemia (n = 17), PD (n = 14), and T2D (n = 12) (Figure 1; Table S1A). Tissue samples were obtained from The Nordic network for Clinical islet Transplantation, supported by the Swedish national strategic research initiative Excellence of Diabetes Research in Sweden. Availability of all five tissues from the same donor was the primary selection criterion in a collection of more than 250 subjects (Figure 1A). The percentage of glycosylated hemoglobin A_1c_ (HbA_1c_) in the blood that indicates the average blood sugar levels over 2–3 months is an established clinical marker of T2D. This and the quantification of insulin secreted from pancreatic islets on stimulation (glucose-stimulated insulin secretion [GSIS]) were strongly associated with the groups of CTRL, PD, and T2D. Anthropometric characteristics, including age, body mass index (BMI), and gender, showed minor associations to HbA_1c_, GSIS, and T2D. Medications administered to subjects during the intensive care hospitalization did not show associations to T2D groups (Table S1B).

In a large proteomics analysis on 195 samples, we identified more than 20,000 proteins (Figures 1A, 1B; Table S1C). Overall, the proteome coverage of tissues was larger than earlier studies, while it overlapped with more than 72% of the proteomes of other studies (Figure 1B; Table S1D).^18^, ^19^, ^20^, ^21^, ^22^, ^23^ One sample and more than 41% of the proteins were excluded from further analysis after a thorough quality-control process (Figures S1A–S1E; Table S1E). Next, we selected as confounding factors clinical and technical variables with median explained variance of proteomics data larger than 1% (Table S1F). Cold ischemic time (CIT), which has been reported to affect the phosphoproteome and potentially the proteome, and the days of hospitalization in the intensive care unit (ICU) were associated to the variance of proteins.^24^ Through the same process, BMI and age in all tissues and the percentage of purity of the sample in pancreatic islets were selected as additional covariates (Table S1F).

Correlation analysis showed that 14, 26, and 27 proteins had strong positive or inverse correlations to Hb_A1c_, GSIS, and BMI, respectively, and retained weak statistical significance after multiple testing correction (Table S1G). The latter was mainly due to the disproportional dimensionality of the data that contain orders of magnitude more proteins than samples. We performed partial correlations of individual proteins to Hb_A1c_ while correcting for covariates including and excluding BMI (Table S1H). Choosing r = |0.4| as the threshold for improved or decreased correlation because of BMI, we concluded that inclusion of BMI as a covariate in the downstream analysis would not influence the associations of proteins to Hb_A1c_. Specifically, the number of proteins correlated to Hb_A1c_ with r > |0.4| remains unchanged after the inclusion of BMI as a confounding factor (Table S1I). Dimensionality reduction using principal-component analysis (PCA) and uniform manifold approximation and projection (UMAP), as well as hierarchical clustering on protein abundancies, showed limited subgrouping of the subjects (Figures 1C, 1D, S1F, and S1G).

### MS proteomics allows accurate clustering of tissues

We performed a stringent quality control of samples and proteins to identify and remove potential outliers and sources of bias (Figures S1A–S1E). Samples from the PD subject p14 were excluded because of consistently deviating more than 3 standard deviations from the overall median of all proteins of the same tissue (Figure S1A), and the dataset was further processed for downstream analysis (Figures 1B–1E and S1B–S1E). We applied a conservative quality-control approach for proteins to be identified in at least 80% of the samples within the same tissue, which resulted in removing more than 40% of the identified proteins across tissues (Table S1E); liver retained more than 4,000 of the originally identified proteins, pancreatic islets and VAT about 3,000, and skeletal muscle more than 1,500 (Figure 1B). As expected, because of the vast dynamic range of the expressed proteome, serum had the lowest number of identified proteins, mainly because of the high abundance of some proteins (Figure 1B; Table S1E).^25^

Despite the known challenges of MS proteomics in VAT and skeletal muscle, our approach resulted in an extremely deep coverage of the proteome of all tissue samples. We also achieved substantial representation of the proteome of pancreatic islets that to date lacks extensive investigation (Table S1D).^26^^,^^27^ Proteins identified in single tissues showed strong enrichment for biological pathways and Gene Ontology (GO) biological processes highly relevant to the corresponding tissues (Figures 1B and S2A). Proteins identified only in liver showed enrichment for metabolism of various types of lipids and amino acids (q < 0.05), transport of bile acids and salts (q < 6 × 10^−4^), and steroid biosynthesis (q < 0.03). Skeletal muscle was enriched for muscle-specific terms, including muscle contraction (q < 8 × 10^−3^) and development (q < 0.03), as well as ion homeostasis (q < 7 × 10^−3^), while pancreatic islets were primarily enriched for secretion of hormones (q < 0.01) and insulin (q < 0.04) and release of signals (q < 3 × 10^−3^), and serum was enriched for the complement and coagulation cascades (q < 0.05) (Figure S2A; Table S1J).

Overall, a set of 1,101 proteins was identified in all tissues excluding serum (Figure 1B), resulting in a collection of more than 600 enriched GO biological processes (q < 0.05) (Figures S2B and S1C; Table S1K). The 87 proteins that were identified across all five tissues resulted in many enriched GO biological processes, despite other larger collections of proteins identified among other subsets of tissues (Figures S2B and S2C). Overall, as expected, proteins identified in two or more tissues represented more generic biological functions than the ones identified in single tissues, including post-translational protein modifications (q < 10^−5^), exocytosis (q < 0.01), generation of precursor metabolites and energy (q < 6 × 10^−17^), tissue homeostasis (q < 2 × 10^−5^), and regulation of cell-cycle G2/M phase transition (q < 9.6 × 10^−12^) (Figure S2C; Table S1K).

Unsupervised clustering revealed identical proteomics profiles for tissue samples from subjects of the current set that were analyzed in a pilot study following a different MS acquisition mode, confirming the robustness of the analysis and the high quality of the data (Figure S3). Furthermore, hierarchical clustering and PCA of protein abundancies showed that the tissues of origin carried the strongest biological signature, over the clinical subgrouping of subjects into CTRL, PD, and T2D (Figures 1C and 1E). The most abundant proteins of the muscle sample p42 that clustered with VAT (Figures 1C and S4A) were related to coagulation and immune response and were similar to the ones from a subset of VAT samples (Figures S4B and S4C), while others that were more similar to skeletal muscle expressed muscle-specific signals (Figures S4B and S4D), collectively suggesting contamination of the sample.

### Landscape of altered biological processes in PD and T2D

**Figure 2:**
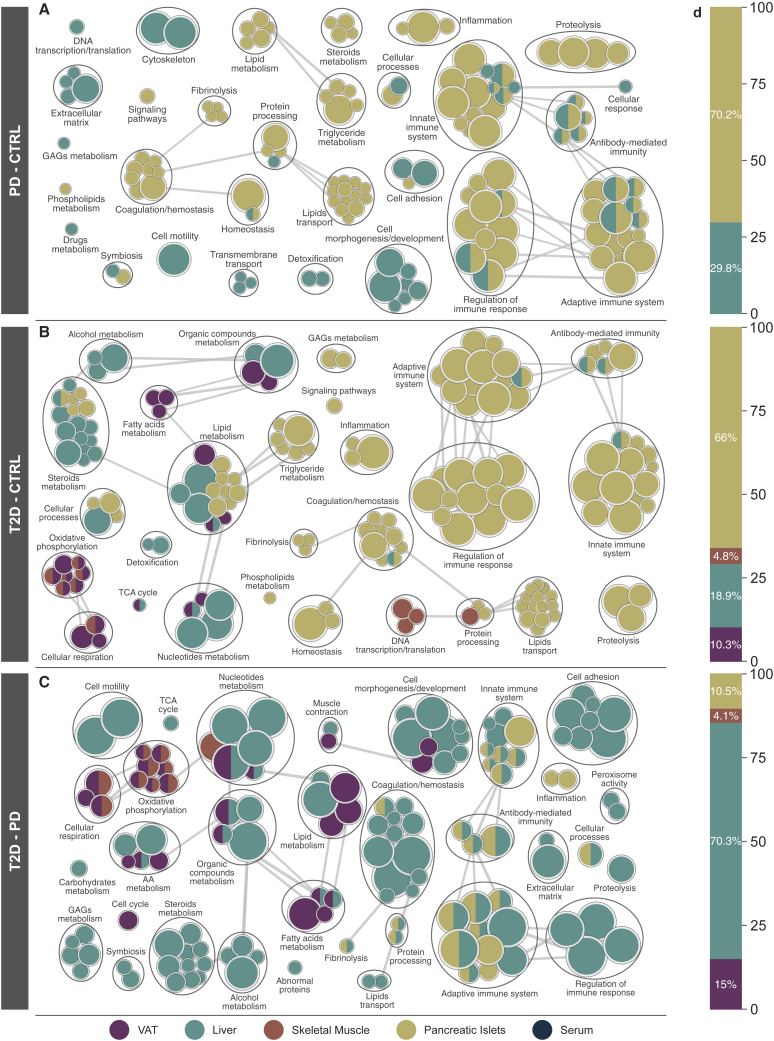
Overview of enriched GO biological processes across tissues for pairwise comparisons among CTRL, PD, and T2D (A–C) Networks were created using the *Cytoscape* plugins *EnrichmentMap* and *AutoAnnotate* based on a curated set of custom classes for GO biological processes (Table S1M).^28^, ^29^, ^30^ Nodes represent enriched GO biological processes (q < 0.01), and their size indicates the number of proteins in the biological process. Thickness of edges represents the fraction of shared proteins between nodes. Only edges describing high similarity were retained. Groups of nodes represent broader categories of individual GO terms. Panels show comparisons of (A) PD-CTRL, (B) T2D-CTRL, and (C) T2D-PD. (D) Distribution of the enriched GO biological processes across tissues corresponding to the networks on the left-hand side. The distribution is presented as the fraction of enriched terms with respect to the total number of enriched terms for the pairwise comparison.

We performed within-tissue differential analyses for pairwise combinations of CTRL, PD, and T2D while accounting for BMI, age, CIT, days of hospitalization in the ICU, and when applicable, percentage of purity of the pancreatic islets sample (Table S1L). Correlation analysis between Spearman r of proteins to Hb_A1c_ (Table S1G) and the log-fold change divided by the standard error from the differential analysis (Table S1L) resulted in r = 0.81 (p < 10^−260^) in liver, r = 0.65 (p < 10^−260^) in pancreatic islets, r = 0.7 (p < 2.3 × 10^−223^) in skeletal muscle, r = 0.73 (p < 10^−260^) in VAT, and r = 0.52 (p < 3.6 × 10^−20^) in serum. In other words, the results of the differential analysis showed significant agreement to the correlation analysis between proteins and Hb_A1c_. Next, we performed a gene set enrichment analysis to explore the landscape of enriched GO terms across tissues and identify altered biological processes across T2D phenotypes^28^, ^29^, ^30^ (Table S1M). When comparing PD and CTRL, the majority of enriched GO terms occurred in pancreatic islets, while the other tissues had a limited number of significantly altered biological processes, even though a considerable number was enriched in the liver (Figure 2A). The dominance of enriched biological processes in pancreatic islets was slightly reduced when T2D was compared with CTRL (Figure 2B). At the same time, we observed multiple enriched GO terms in skeletal muscle and VAT (Figure 2D). Pancreatic islets showed a limited number of enriched terms when T2D was compared with PD (10%), while over 70% of the same enrichment terms were covered by the liver (Figures 2C and 2D). A similar fraction of enriched terms for skeletal muscle was observed in the T2D-CTRL and T2D-PD networks, while for liver and VAT the largest number of enriched processes was observed in the T2D-PD network, 70% and 15%, respectively (Figures 2B and 2C). In contrast, pancreatic islets were responsible for over 66% of the enriched GO terms in both PD-CTRL and T2D-CTRL (Figures 2A, 2B, and 2D). Overall, the distribution of clusters of GO biological process across tissues varied for different combinations of CTRL, PD, and T2D (Figure S6A). VAT, muscle, and pancreatic islets demonstrated enriched GO terms covering similar biological processes, while the liver projected an intermediate state with more diversely enriched groups. Across tissues, pairwise comparison of liver and pancreatic islets, as well as VAT and skeletal muscle, demonstrated large similarities on the patterns of enriched GO terms (Figure S6B).

### Immune and lipid-related processes are upregulated in pancreatic islets of PD

The majority of enriched GO biological processes related to the immune system and its regulation in pancreatic islets were significantly upregulated in PD (q_max_ < 3.7 × 10^−3^). Processes related to adaptive and innate immune systems, antibody-mediated immunity, and inflammation were consistently elevated in pancreatic islets (q_max_ < 7.1 × 10^−4^), as well as in liver (q_max_ < 5 × 10^−3^) of PD. Moreover, terms related to coagulation and homeostasis were also higher in PD of pancreatic islets (q_max_ < 5.8 × 10^−3^). Homeostasis was significantly altered in pancreatic islets of PD (q_max_ < 2.5 × 10^−3^), both on the fibrinolytic and on the coagulation end, which when taken together with the elevated immune responses create the foundation of a pre-thrombotic state.^31^ Lipid metabolism pathways, including metabolism of steroids and triglycerides (q_max_ < 1.2 × 10^−3^), and metabolism of lipids, as well as their transport (q_max_ < 2.5 × 10^−3^), were significantly increased in PD of pancreatic islets (Figures 2A and S5A).

### Alteration of biological processes in VAT and skeletal muscle of T2D

Biological processes connected to metabolism of steroids and alcohol were almost exclusively upregulated in liver of T2D (q_max_ < 8.4 × 10^−3^), while those associated with proteolysis increased in pancreatic islets of T2D (q_max_ < 4.7 × 10^−3^). We observed divergent directions in the alterations of the metabolism of organic compounds and nucleotides between liver (q_max_ < 6.7 × 10^−3^) and VAT of T2D (q_max_ < 4.8 × 10^−3^). We also identified a notable decrease in GO terms related to oxidative phosphorylation (OXPHOS) and cellular respiration in VAT (q_max_ < 4.1 × 10^−3^) and skeletal muscle (q_max_ < 3.4 × 10^−3^) (Figures 2B and S5B). The latter confirms our observation that enriched GO terms in VAT and muscle share a considerable fraction of ontology terms in T2D (Figure S6B). Various processes related to the metabolism of fatty acids were exclusively decreased in VAT of T2D (q_max_ < 4.2 × 10^−3^), while skeletal muscle showed a significant decrease for terms related to DNA transcription and translation in T2D (q_max_ < 5.3 × 10^−3^) (Figure 2B).

### Deregulation of various biological processes from PD to T2D in the liver

Comparison of T2D and PD resulted in the largest number of enriched biological processes across tissues, with liver being responsible for over 70% of them and pancreatic islets for less than 11% (Figure 2D). Immune-related terms and their regulatory processes largely occurred in liver (q_max_ < 6.3 × 10^−3^) that consists of a shift in comparison with the former networks that compared PD and T2D with CTRL and that were dominated by pancreatic islets (Figures 2C and S6A). This had 2-fold implications on the similarity of inflammatory and immune states between PD and T2D in pancreatic islets and on the late alterations of these biological processes in liver in T2D (Figure S5C). Multiple enriched metabolic processes were exclusively altered in liver, probably because of its higher metabolic activity spanning various metabolic processes (q_max_ < 8.4 × 10^−3^). Specifically, metabolic processes of carbohydrates (q_max_ < 8.3 × 10^−3^), alcohol (q_max_ < 1.4 × 10^−4^), and steroids (q_max_ < 1.3 × 10^−3^) were upregulated, whereas metabolism of glycosaminoglycans was downregulated (q_max_ < 8.4 × 10^−3^) (Figure 2C). Metabolic processes of amino acids (q_max_ < 8.2 × 10^−3^), organic compounds (q_max_ < 3.2 × 10^−4^), fatty acids (q_max_ < 3.3 × 10^−3^), and lipids (q_max_ < 7.7 × 10^−3^) were consistently upregulated in liver and downregulated in VAT (q_max_ < 2.2 × 10^−4^, q_max_ < 1.6 × 10^−7^, q_max_ < 2.1 × 10^−3^, and q_max_ < 2.8 × 10^−3^, respectively) of T2D in comparison with PD (Figure 2C). Similar to T2D-CTRL, OXPHOS and cellular respiration were enriched in muscle (q_max_ < 3.1 × 10^−4^ and q_max_ < 3.3 × 10^−5^, respectively), as well as VAT (q_max_ < 6.4 × 10^−4^ and q_max_ < 6.9 × 10^−5^, respectively), and followed the same direction of change (Figures 2C and S5C). Overall, biological processes enriched in the comparison T2D-PD resembled those enriched in T2D-CTRL in muscle and VAT (Figures 2B, 2C, and S6B). The latter did not apply to the same extent in liver, where about half of the biological processes were shared across comparisons (Figure S6B).

### Enrichment of key metabolic pathways across tissues in PD and T2D

**Figure 3:**
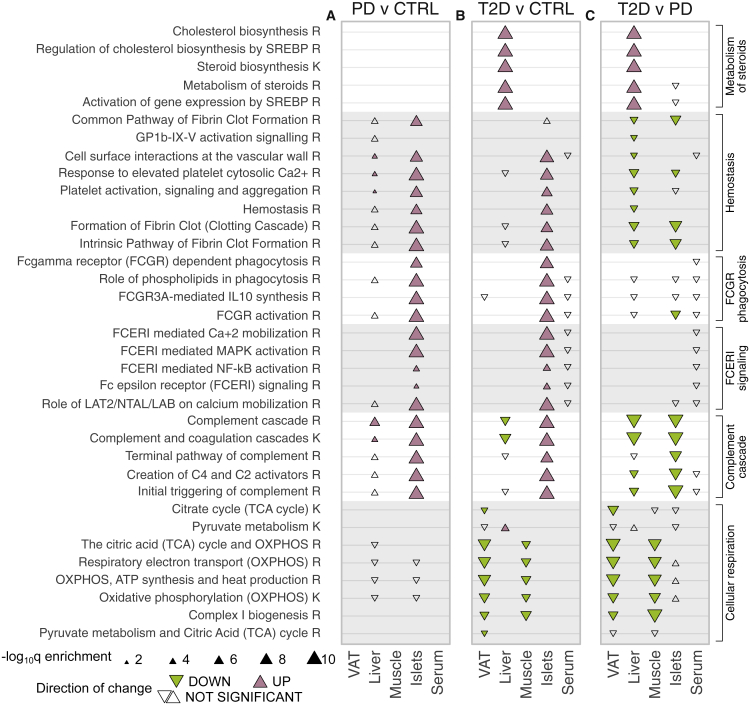
Significantly enriched biological pathways (A–C) Selected groups of enriched biological pathways (q ≤ 0.5) for pairwise comparisons among CTRL, PD, and T2D, specifically, (A) PD-CTRL, (B) T2D-CTRL, and (C) T2D-PD. Colored triangles indicate statistical significance (q < 0.05) and direction. Rows represent pathways, panels represent pairwise comparisons, and columns within panels represent tissues. The database of origin for each pathway is noted with a suffix R or K for Reactome or Kyoto Encyclopedia of Genes and Genomes (KEGG), respectively. GP1b-IX-V, glycoprotein Ib-IX-V; SREBP, sterol regulatory element binding protein.

We analyzed the dataset for enriched biological pathways similarly to the aforementioned analysis for GO terms (Table S1N). The fraction of enriched biological pathways per tissue (q < 0.05) followed the same pattern as the GO biological processes (Figures 2D and S6C). We selected a subset of enriched biological pathways (q < 0.05) that we grouped based on biological relevance (Figure 3). The TCA and its contributions of NADH and FADH_2_ electron carriers to the OXPHOS have been observed to be decreased in muscle of T2D and in adipose tissue of insulin-resistant subjects.^32^, ^33^, ^34^ Here we observed that the entirety of TCA and OXPHOS were significantly decreased in T2D of VAT and muscle when compared with both CTRL and PD, as well as the merged group of CTRL + PD (Figures 3B, 3C, and S7B). At the same time, pathways related to the complement cascade were significantly increased in PD of liver and pancreatic islets (Figure 3A). In liver of T2D, some of these pathways were significantly lower when compared with both CTRL and PD, suggesting widespread inflammation in PD and the inverse in T2D (Figure S7B). Various resolution levels for biological pathways related to Fc-γ and Fc-ε signaling, as well as hemostasis, were significantly higher in PD and T2D in pancreatic islets, with the ones of T2D being significantly lower than the ones in PD (Figure 3). The latter is further confirmed from the statistical significance between the merged group of PD and T2D to CTRL (Figure S7A; Table S1N) and the lack of significance when PD is considered in combination with CTRL and compared with T2D (Figure S7B; Table S1N). Metabolism of steroids enclosed five different pathways that were all significantly higher in liver of T2D compared with CTRL, while differences between PD and CTRL did not show significance (Figure 3). The similarity of pathways related to metabolism of steroids in liver of PD and CTRL subjects is also demonstrated when they are merged into one non-diabetes group and compared with T2D (Figure S7B). Other enriched pathways included the increase of metabolism of vitamins in pancreatic islets of PD and T2D and the decrease of pathways of neurodegenerative diseases in VAT and liver of T2D (Table S1N).

We also investigated whether the insulin doses that were administered to three subjects from a CTRL while in ICU affected our results (Table S1A). Specifically, we excluded those subjects and reperformed the enrichment analysis of biological pathways and GO terms. Comparison of the significance levels and the normalized enrichment scores between this analysis and the original results showed negligible differences (Figure S8; Table S1O).

### TCA and OXPHOS in VAT and skeletal muscle are significantly decreased in T2D

**Figure 4:**
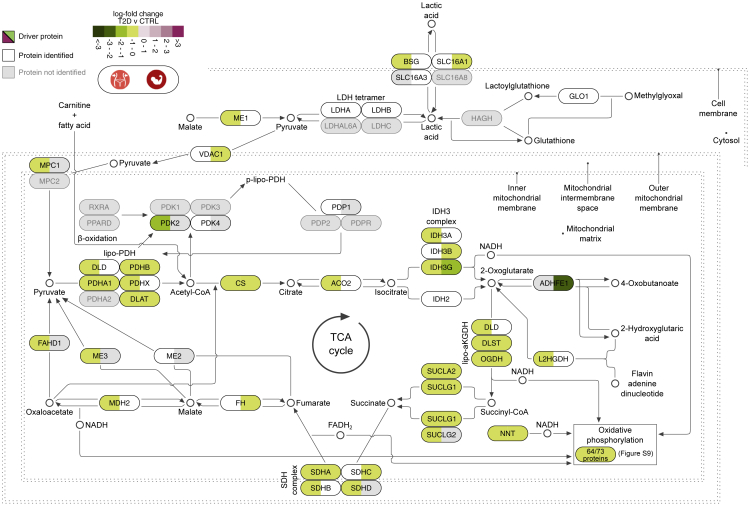
Biological pathway of pyruvate metabolism and the TCA cycle Pathway visualization based on Reactome and complemented with details from KEGG and the literature. Tiles illustrate log-fold change values of T2D compared with CTRL in VAT (left-hand side of tile) and skeletal muscle (right-hand side of tile). Proteins leading the decrease of the pathway are marked as driving and are shown in black font and colored tiles. Proteins participating in the pathway and identified from MS proteomics, but not marked as leading edges, are in white tiles, whereas those in gray were not identified in this analysis.

Mitochondrial function reflected by the TCA cycle and the OXPHOS chain were impaired in T2D in comparison with both CTRL and PD individuals (Figure 4). The same was also found for the biogenesis of the key mitochondrial component complex I, which is the leading component of the reduction in OXPHOS in skeletal muscle and VAT. In contrast, there were no evident differences in these pathways between PD and CTRL subjects (Figures 3A, S9C, and S9D). Analysis of mitochondrion-related GO cellular components revealed significant downregulation for VAT and skeletal muscle for several terms, including mitochondrial matrix (q_VAT_ < 1.1 × 10^−5^ and q_muscle_ < 1.3 × 10^−2^), inner mitochondrial membrane protein complex (q_VAT_ < 7.2 × 10^−6^ and q_muscle_ < 1.7 × 10^−5^), mitochondrial envelope (q_VAT_ < 2.7 × 10^−6^ and q_muscle_ < 9 × 10^−5^), and mitochondrial protein complex (q_VAT_ < 10^−6^ and q_muscle_ < 1.7 × 10^−5^) (Table S1P).

The entirety of the TCA cycle and the OXPHOS chain was significantly lower in T2D than in CTRL in VAT and skeletal muscle (q < 8.8 × 10^−8^ and q < 3.2 × 10^−4^, respectively) (Figure 3; Table S1N). The downregulation of the TCA cycle in VAT and muscle has been described in other studies to be associated with IR.^32^, ^33^, ^34^ The downregulation of pyruvate metabolism and the TCA cycle was driven by 26 proteins in VAT and 17 proteins in skeletal muscle, of which 11 were shared (Figure S9). The vast majority of all eight key enzymatic complexes of the TCA cycle, namely, citrate synthase (CS), aconitase 2 (ACO2), isocitrate dehydrogenase (IDH), α-ketoglutarate dehydrogenase (αKGDH), succinyl-CoA synthase (SCS), succinate dehydrogenase (SDH), fumarate hydratase (FH), and malate dehydrogenase (MDH), were reduced in VAT and/or skeletal muscle of T2D, hence confirming the significant downregulation of the entire pathway. The NADH and FADH_2_ electron carriers produced from the TCA cycle are subsequently imported to the OXPHOS and utilized for energy production (Figure 4). A large set of 54 proteins from VAT and 27 from skeletal muscle, of which 19 were shared, were marked to drive the overall decrease of the OXPHOS pathway components (Figures 3 and S10). The majority of the driving proteins were moderately higher in PD than in CTRL, but their notable decrease in T2D resulted in the significant decrease of the pathway in both VAT and muscle. In conclusion, we confirm previous knowledge about the downregulation of TCA and OXPHOS in VAT and skeletal muscle in T2D. Furthermore, we extend this knowledge by identifying additional deregulated proteins, which highlights the value of the data.

### Cholesterol biosynthesis is significantly increased in liver of T2D

**Figure 5:**
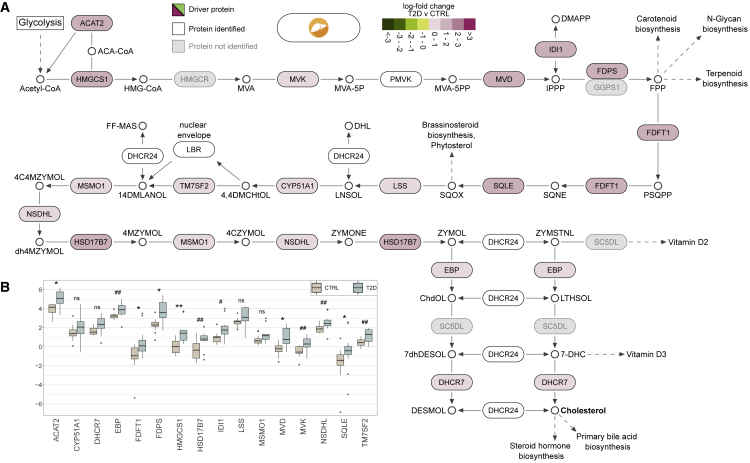
Detail on the increase of the biological pathway of cholesterol biosynthesis in liver (A) Pathway visualization based on Reactome and complemented with details from KEGG and the literature. Tiles illustrate log-fold change values of T2D compared with CTRL in liver. Proteins leading the increase of the pathway are marked as driving and are shown in black font and colored tiles. Proteins participating in the pathway and identified from MS proteomics, but not marked as leading edges, are in white tiles, whereas those in gray were not identified in this analysis. (B) Detailed comparison of the abundance of driving proteins between T2D and CTRL in liver.

Multiple steps of the cholesterol biosynthesis pathway were enhanced in liver of T2D compared with PD, as well as CTRL (Figures 3, 5, S7A, and S7B). The increase in liver of T2D subjects (q < 1.6 × 10^−56^) was mainly driven by 16 proteins that demonstrated up to 2-fold higher levels when compared with CTRL (Figure 5; Table S1L). Eleven of the 16 proteins are products of the biological pathway that activates gene expression through sterol regulatory element binding proteins (SREBPs) (Figures 3 and 5). Specifically, the expression of, among others, lanosterol demethylase (CYP51A1), 7-dehydrocholesterol reductase (DHCR7), farnesyldiphosphate farnesyltransferase (FDFT1), farnesyl diphosphate synthase (FDPS), hydroxymethylglutaryl CoA synthase (HMGCS1), isopentenyl-diphosphate δ-isomerase 1 (IDI1), lanosterol synthase (LSS), diphosphomevalonate decarboxylase (MVD), mevalonate kinase (MVK), squalene monooxygenase (SQLE), and transmembrane 7 superfamily member 2 (TM7SF2) was increased 1- to 2-fold in liver of T2D compared with CTRL (Figure 5B).

The cholesterol biosynthesis pathway initiates with acetyl-CoA that is produced by glycolysis and results in, among others, cholesterol that is utilized for the biosynthesis of steroid hormones and primary bile acids. The MS analysis identified most of the proteins from key steps of the cholesterol biosynthesis pathway (Figure 5A). A detailed exploration on the protein abundancies in liver revealed that despite the moderate significance of the differences between CTRL and T2D in protein levels, their cumulative impact to the pathway was sufficient to lead to a strong increase (Figures 5B and S11).

### Proteins related to the hemostasis pathway were elevated in pancreatic islets of PD

**Figure 6:**
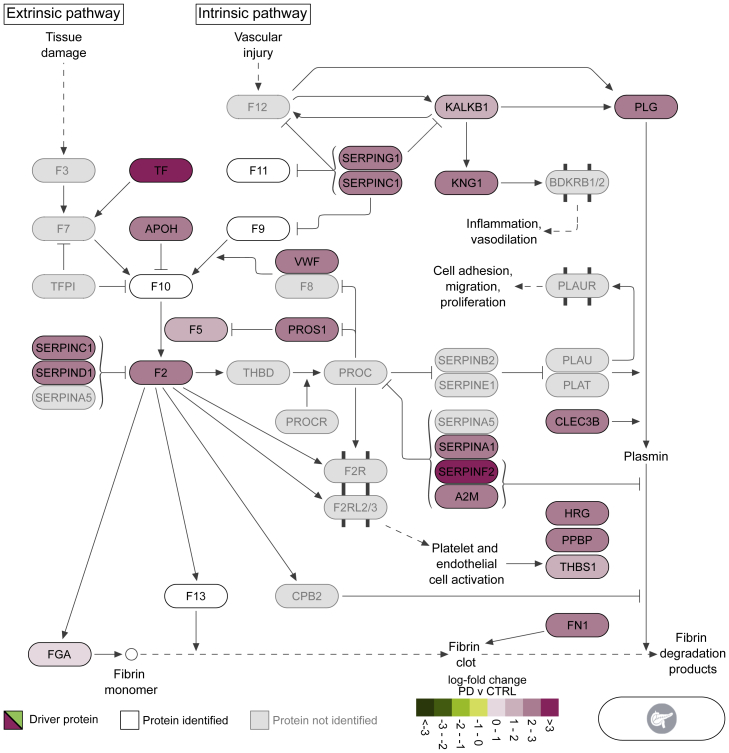
Biological pathway of hemostasis (coagulation cascade) Pathway visualization based on Reactome and complemented with details from KEGG and the literature. Tiles illustrate log-fold change values of PD compared with CTRL in pancreatic islets. Proteins leading the increase of the pathway are marked as driving and are shown in black font and colored tiles. Proteins participating in the pathway and identified from MS proteomics, but not marked as leading edges, are in white tiles, whereas those in gray were not identified in this analysis.

The blood clot formation pathway of hemostasis was strongly enriched in pancreatic islets of PD (q < 3.5 × 10^−6^) (Figure 3). Specifically, the enrichment of the clotting cascade (q < 8.6 × 10^−14^) was led by the upregulation of the common and intrinsic pathways that are responsible for fibrin clot formations (q < 1.1 × 10^−6^ and q < 4.5 × 10^−15^, respectively) (Figure 3; Table S1N). A large collection of 44 proteins that were higher in PD than in CTRL drove the identification of the significant increase of the pathway (Figures S12). Visualization of a subset of 21 proteins from the pathway of hemostasis illustrated the key biological processes affected by these enzymes (Figure 6). We observed a greater than 3-fold increase in transferrin (TF) and a solid elevation in multiple members of the serpin family of proteins (Figures 6 and S12A). In addition, we observed that the significant increase of the 44 proteins associated with hemostasis was followed by a significant decrease in T2D that, in turn, was significantly higher than CTRL (Figure S12B). In contrast with islets, hemostasis-related pathways in liver were significantly lower in T2D than in PD (Figure 3).

### Complement cascade increases in liver and pancreatic islets of PD

**Figure 7:**
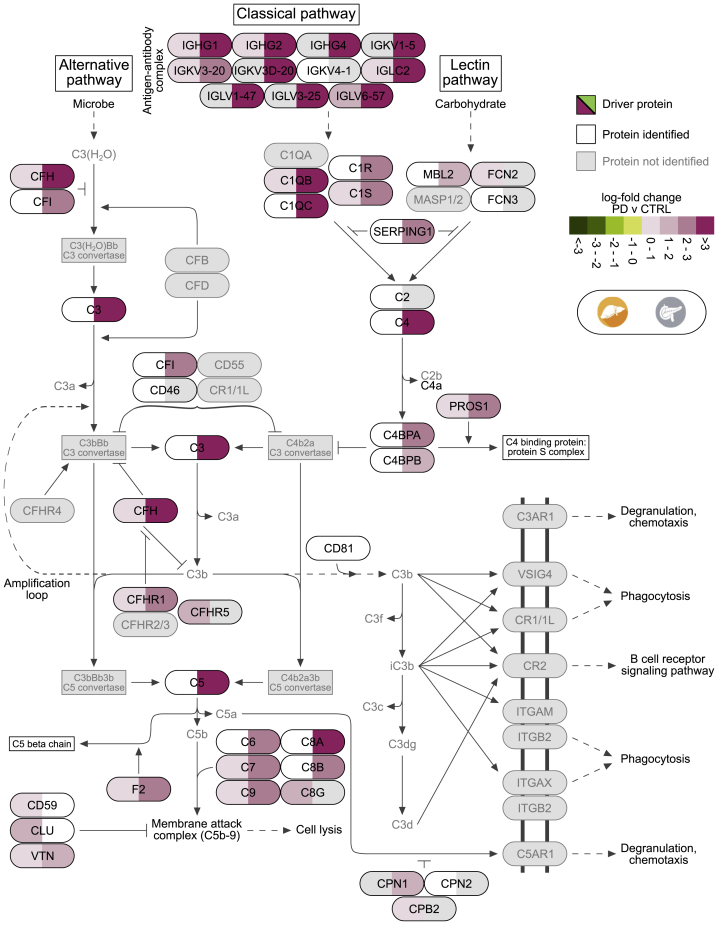
Biological pathway of complement cascade Pathway visualization based on Reactome and complemented with details from KEGG and the literature. Tiles illustrate log-fold change values of PD compared with CTRL in liver (left-hand side of tile) and pancreatic islets (right-hand side of tile). Proteins leading the increase of the pathway are marked as driving and are shown in black font and colored tiles. Proteins participating in the pathway and identified from MS proteomics, but not marked as leading edges, are in white tiles, while those in gray were not identified in this analysis.

The complement cascade was significantly higher in PD than CTRL in liver and pancreatic islets (q < 8.4 × 10^−5^ and q < 1.6 × 10^−72^, respectively) (Figure 3; Table S1N). In detail, the classical and lectin pathways, which are responsible for the creation of the C2 and C4 activators, were strongly elevated in pancreatic islets (q < 2.8 × 10^−53^) but did not cross the threshold of statistical significance in liver (Figures S13A and S13B). The upregulation of the pathway in liver was led by 20 proteins, while the one in pancreatic islets by 34 proteins, of which 14 were shared (Figures 7, S13A, and S13B). The complement cascade remained higher in T2D than CTRL in pancreatic islets, in contrast with liver, where it significantly decreased (Figure 3). The two tissues were in agreement when comparing T2D with PD. Specifically, the levels of the driving proteins for the upregulation of the complement cascade in PD decreased in T2D, resulting in an overall downregulation of the pathway from PD to T2D (q_liver_ < 1.4 × 10^−14^ and q_islets_ < 1.1 × 10^−16^) (Figures 3, S13C, and S13D). The latter clearly marked an abrupt increase of immune system-related proteins in PD followed by a strong decrease in T2D that in liver led to significantly higher levels in T2D compared with CTRL (Figures S13C and S13D).

## Discussion

We performed a MS-based proteomics analysis of samples from VAT, liver, skeletal muscle, pancreatic islets, and serum from a cohort of 43 multi-organ donors (Figure 1). We provided the deepest proteome coverage to date for multiple tissues, including the poorly studied pancreatic islets (Table S1D) coupled to a thorough conservative quality-control process to ensure the robustness of the results (Figures S1, S3, S4, and S8; Tables S1D–S1I and S1O). We performed a multi-dimensional exploratory analysis that highlighted biological processes and pathways altered along with the development of T2D (Figures 2 and 3). This map of defects in pathways was complemented with a fine-grained collection of proteins that were shown to drive their direction of change, as well as their magnitude (Figures 4, 5, 6, and 7). Our findings highlighted several biological pathways to be significantly altered in PD and T2D and, most importantly, paved the way for further intra-individual investigations. Overall, our study provides a resource to the field of metabolic diseases.

It is estimated that 70%–80% of the patients with T2D have non-alcoholic fatty liver disease (NAFLD).^35^^,^^36^ We compared our results with recent studies that have investigated proteomics for NAFLD in tissues difficult to sample, such as liver and pancreatic islets. The slight decrease of fructose-bisphosphate aldolase B (ALDOB) in T2D of pancreatic islets was not statistically significant, hence it cannot contradict recent findings from Wigger et al.^37^ ALDOA was unchanged in all tissues in agreement with their findings,^37^ while ALDOB and ALDOC were increased in T2D of liver (Table S1L). Upregulation of ALDOB in liver has been associated with steatosis in subjects with alcohol-related liver disease (ALD) and NAFLD.^21^^,^^22^ In contrast, we observed upregulation of glyoxalase I (GLO1) in liver of PD and T2D, the downregulation of which in the same tissue of murine models has been associated with NAFLD.^38^

The vast majority of the biological processes that were enriched in PD and T2D of pancreatic islets and liver were related to immune responses and their regulation (Figures 3A, 3B, S5A, and S5B). Links between the activation of the adaptive and the innate immune system and the progression of T2D have been demonstrated in earlier studies, while other proteomics studies have shown a strong increase in immune response in human islets of T2D subjects.^39^, ^40^, ^41^ The most interesting group in the PD network was the upregulation of biological processes involved in the regulation of the immune response in pancreatic islets and liver (Figures 2A and 5A). Liver also showed divergent patterns of alterations in PD and T2D, while in VAT and skeletal muscle there was a prevalent decrease in T2D (Figures 2A, 2B, S5A, and S5B). Comparison between T2D and PD not only provided insights for the tissues with strong responses to T2D but also underlined significantly affected biological processes (Figure 2C). We observed no enriched biological processes or pathways in PD of VAT and skeletal muscle, while we identified very few differences between PD and T2D in pancreatic islets. In general, pancreatic islets dominated the alterations between PD and CTRL, and in T2D they did not demonstrate further notable alterations (Figure 2). Unlike pancreatic islets, in liver there were over 150 enriched biological processes mainly related to the immune system that differed between PD and T2D (Figure 2C). In summary, our data suggest that most of the biological alterations in PD occur primarily in pancreatic islets and secondarily in liver, and in T2D, alterations related to OXPHOS and cellular respiration become prominent in VAT and skeletal muscle (Figure 2).

The TCA cycle has previously been suggested to be a marker of IR in various tissues that we, in agreement with other studies, find to be strongly downregulated in T2D of skeletal muscle and VAT (Figures 3 and 4).^32^, ^33^, ^34^ Specifically, other proteomics studies have shown the TCA, together with OXPHOS and pyruvate metabolism, to be significantly downregulated in VAT.^20^ The amphibolic nature of the pathway involves the synthesis of fatty acids and amino acids, besides driving synthesis of ATP from ADP. In other words, its overall downregulation will affect the ATP production and other important metabolic pathways.^42^ The pivotal role of mitochondrial OXPHOS in T2D was highlighted in a recent study that successfully treated T2D in mouse models using magnetic and electric fields.^43^ The impairment of the TCA cycle and the OXPHOS chain in VAT and skeletal muscle of T2D subjects compared with CTRL and PD, in combination with the indistinguishable differences between CTRL and PD, suggested a relatively late event becoming evident when diabetes is already established, rather than during its development (Figure 3). Taken together, our data suggested the tissues and the T2D stage at which the downregulation of the TCA cycle and the OXPHOS chain become prominent.

A similar sequence of events applied to the enhanced cholesterol biosynthesis in liver (Figures 3 and 5). This may be driven by the enhanced transcription activation of genes encoding various key enzymes of the pathway and may be linked to subsequent dysregulation of cholesterol and lipoprotein production (Figures 3 and 5). This also became evident in T2D and is likely to be a consequence rather than a cause of the disease. The elevated level of many enzymes of cholesterol biosynthesis was likely driven by two biological pathways that have SREBP as their core regulator, namely, regulation of cholesterol biosynthesis by SREBP and gene expression activation by SREBP (Figure 3). We hypothesize that it is driven by increased levels and activity of SREBP transcription factors. The latter may subsequently lead to increased synthesis or assembly of other lipid moieties, such as triacylglycerols, and it may favor the production and release into the circulation of lipoproteins, such as very-low-density lipoprotein particles.^44^^,^^45^

Liver, skeletal muscle, and VAT are the classical sites for insulin-regulated substrate metabolism, accounting for much of energy expenditure and storage.^46^^,^^47^ We identified patterns of enhanced lipid, in particular cholesterol, synthesis in the liver, and reduced mitochondrial function, and hence glucose and lipid oxidation, in VAT and skeletal muscle (Figure 3). Taken together, these alterations are compatible with less utilization of energy substrates in muscle and adipose tissue, redistribution of lipids toward the liver, and altered lipoprotein release. It appears that these alterations in energy metabolism occur later than the islet perturbations, and in fact, they can partly be secondary to a relative insulin deficiency. Nonetheless, the alterations in liver, muscle, and VAT are certainly important in maintaining the diabetic state, including hyperglycemia, dyslipidemia, and overweight. The established endocrine dysfunction of the pancreas and adaptative mechanisms of the brain are also involved in “defending” chronic hyperglycemia in T2D.^9^^,^^48^^,^^49^ At the same time, the liver, primarily, and to a lesser extent, the kidneys are responsible for gluconeogenesis that is the major endogenous source of circulating glucose. It is well known that elevated gluconeogenesis is among the main drivers of hyperglycemia in T2D, and the present findings suggested significantly impaired mitochondrial function, which, in turn, can promote gluconeogenesis.^50^

IR with compensatory hyperinsulinemia is a common feature of T2D and impaired glucose tolerance, both of which are associated with increased risk for coronary heart disease (CHD).^51^ A partial explanation for the link between IR and CHD can be found in the hypercoagulable state that is characteristic of individuals with PD or T2D.^52^ Some studies have indicated a connection between hyperinsulinemia/hyperglycemia and activation of the coagulation cascade following food intake,^53^^,^^54^ while others have shown a general increase in the risk for venous thromboembolism in patients with T2D.^55^ These findings have been further supported by reports that infusion of glucose induces a transient increase in the generation of thrombin (F2) in healthy subjects and of the effect being more pronounced in T2D patients. In addition, animal studies have shown that obesity can lead to increased F2 production in adipose tissue via increased F7 activity; conversely, F2 deletion may protect from adiposity and IR.^56^^,^^57^ Here, we observed strong elevation in the levels of TF, F2, and several members of the serpin family, including PAI-1, that play important roles in formation and resolution of fibrin clots, and this is thus expected to result in a net prothrombotic effect (Figure 6). Moreover, multiple serine proteases of the coagulation system have been shown to function as alternative activators of C5 and C3 of the complement cascade,^58^ which was observed to be significantly elevated in other proteomic analyses of plasma from patients with T2D.^59^^,^^60^ These pathways were strongly upregulated in PD of pancreatic islets, and the latter was also upregulated in liver of PD (Figures 3, 6, and 7).

It is of great interest that pancreatic islets, in contrast with liver, VAT, and skeletal muscle, displayed strong perturbations of protein patterns in PD individuals compared with CTRLs (Figures 2 and 3). Evidence for increased hemostatic and inflammatory activity in PD was not further enhanced in T2D subjects (Figure 3). Instead, they retained similar patterns to PD for immune responses, while there was even a partial return toward the levels of CTRL with respect to complement activation and hemostasis via platelet and coagulation activation (Figures S12B, S13C, and S13D). In conclusion, these data are compatible with an early, potentially causal role of vascular, inflammatory, and immune impairment within the pancreatic islets that lead to dysfunction of their endocrine cells, which may be manifested by insufficient insulin production and increased glucagon levels.^9^^,^^37^^,^^61^ It could therefore be speculated that there is a vascular/inflammatory insulitis to pancreatic islets that occurs mainly during diabetes development but later on subsides.

In conclusion, we reported an extensive collection of biological pathways that were found to be altered across several metabolically relevant tissues in the development of T2D. More importantly, we presented a multi-tissue proteomics map describing the tissue-specific metabolic dysregulation across healthy, PD, and T2D subjects.

### Limitations of the study

Subjects enrolled in this study were treated with multiple medications in the ICU, often including glucocorticoids and insulin, which might interact with the underlying metabolic condition to modulate the proteomic data. In ICU patients, temporary hyperglycemia commonly occurs, also in non-diabetic patients, thus patients without diabetes, and obviously those with diabetes, are often given insulin to maintain near normoglycemia. Patients who were not administered insulin maintain an endogenous level of insulin produced by their β cells. Taken together, this indicates that our observed differences in protein profiles between groups are likely related to the chronic glycemic status and not to acute glycemic alterations during ICU care.

Our results would need to be cross-validated in other settings involving additional experimental procedures to be further confirmed and, potentially, explored in clinical studies. We were restricted only to the reported clinical variables because of the Swedish legislation on obtaining details for other underlying diseases and medication that the subjects were administered prior to their admission to the ICU. In our analysis, we have adjusted for potential confounding technical factors including CIT and the purity of the samples of pancreatic islets. The nature of this study that includes factors such as underlying inflammation in subjects and the unavailability of clinical variables might have impacted a fraction of the results. The choice of serum limited us from investigating coagulation factors in blood. In addition, sampling methods at the different medical facilities and the control of deep-frozen storage chain of the samples may have impacted the results to some extent.

## STAR★Methods

### Key resources table

**Table:** 

REAGENT or RESOURCE	SOURCE	IDENTIFIER
**Chemicals, peptides, and recombinant proteins**		

Sodium deoxycholate (SDC)	Sigma	Cat#30970
Tris (2-carboxyethyl) phosphine	Sigma	Cat#C4706
Chloroacetamide	Sigma	Cat#C0267
LysC	Wako	Cat#4548995075888
Trypsin	Promega	Cat#V5111
Acetonitrile	Sigma	Cat#75-05-8
25% LC-MS grade ammonia	Fisher chemical	Cat#533003
Trifluoroacetic acid	Sigma	Cat#76-05-1
Water, Optima L/MS Grade	Sigma	Cat#76-05-1

**Deposited data**		

Proteomics Identifications Database (PRIDE)		PXD027597
Full analysis pipeline	https://github.com/klevdiamanti/multi-tissue_ms_proteomics	

**Software and algorithms**		

Cytoscape v3.7	Shannon et al., 2003	
EnrichmentMap v3.3	Merico et al., 2010	
AutoAnnotate v1.3.3	Kucera et al., 2016	
limma v3.42.2	Ritchie et al., 2015	
Camera	Wu et al., 2012	
fgsea v1.12.0	Korotkevich et al., 2019	
MaxQuant v1.6.0.1	Cox and Mann 2008	
Andromeda	Cox et al., 2011	
Spectronaut v13	Bruderer et al., 2015	

**Other**		

MSigDB v7.2 (19 September 2020)	Liberzon et al., 2011	
UniProt	The UniProt Consortium, 2021	
GO (from MSigDB v7.2)	Subramanian et al., 2005	
GOslim (02 May 2020)	Mi et al., 2019	
KEGG (from MSigDB v7.2)	Kanehisa et al., 2017	
Reactome (from MSigDB v7.2)	Jassal et al., 2020	
Empore SPE SDB-RPS disc	Sigma	Cat#66886-U
Reprosil-Pur Basic C18, 1.9μm	Dr. Maisch Gmbh	Cat#r119.b9
EASY-Nlc 1200 system	Thermo Fisher	Cat#LC140
Q Exactive™ HF-X Hybrid Quadrupole-Orbitrap™ Mass Spectrometer	Thermo Fisher	Cat#IQLAAEGAAPFALGMBFZ

### Resource availability

#### Lead contact

Further information and requests should be directed to and will be fulfilled by the lead contact, Prof. Claes Wadelius (claes.wadelius@igp.uu.se).

#### Materials availability

This study did not generate new unique reagents.

### Experimental model and subject details

#### Ethics declaration

The collection and utilization of human organs for scientific research and transplantation purposes is regulated by the Swedish legislation. The human tissue lab is a biobank for multi-organ donors funded by the excellence of diabetes research in Sweden (EXODIAB) and is a collaborative initiative between the universities of Uppsala and Lund. Samples (n = 43) for five metabolically relevant tissues were obtained from the human tissue lab following the Uppsala Regional Ethics Committee approval (Dnr: 2014/391). Informed consent was received from the donors or their legal guardians for their organs to be used for scientific research. Storage and analysis of the samples has been in full accordance with the Swedish law and regional standard practices. No tissue samples were obtained from prisoners.

#### Sample collection

Frozen samples of VAT, liver, skeletal muscle, pancreatic islets and serum were obtained for 43 multi-organ donors (Tables S1A and S1B). All subjects remained in the ICU mostly for 2–3 days (range 1–12) and brain death was the reported cause of death for all subjects. The world health organization guidelines were followed to identify T2D subjects in the corresponding medical facilities.^63^ The percentage of HbA_1c_ in the blood that indicates the average blood sugar levels over 3–4 weeks was analyzed according to hospital clinical routines established by the department of clinical chemistry at Uppsala university and with full accreditation. The assay is performed on an Afinion instrument from Abbot. PD was identified based on the percentage of glycosylated hemoglobin in blood (5.7% ≤ HbA_1c_≤6.5%), while normoglycemia (CTRL) was assigned otherwise (HbA_1c_≤5.6%) (Tables S1A and S1B).

GSIS was assessed in a dynamic perfusion system, Suprafusion 1000 (BRANDEL, Gaithersburg, MD). Twenty handpicked islets were perfused with low glucose (1.67 mM) for 42 min, high glucose (20 mM) for 48 min, and then low glucose again. Fractions were collected at 6 min intervals and the secreted insulin was measured by enzyme-linked immunosorbent assay (ELISA) (Mercodia, Uppsala, Sweden). In addition, purity of the pancreatic islet samples were estimated.^64^ Information on characteristics of the anthropometric, technical, T2D-related and ICU-related variables was available for the study and was described in (Table S1B). We also explored merging PD with T2D and comparing to CTRL, additionally to merging PD with CTRL and comparing to T2D.

### Method details

#### Mass spectrometry proteomics

##### Proteomic sample preparation

Skeletal muscle, VAT and liver samples were lysed in Sodium Deoxycholate containing lysis buffer (1% Sodium Deoxycholate (SDC), 10 mM Tris (2-carboxyethyl) phosphine (TCEP), 40 mM Chloroacetamide (CAA) and 100 mM of Tris (pH 8.5)). The samples were homogenized with an Ultra Turrax homogenizer (IKA). In case of the pancreatic islets extract, lysis buffer containing 10% 2,2,2-trifluoroethanol (TFE), 10 mM Tris (2-carboxyethyl) phosphine (TCEP), 40 mM Chloroacetamide (CAA) and 100 mM of Tris (pH 8.5) was used for protein extraction. For the serum samples, 1μL of serum was mixed with 24 μL of 1% SDC buffer. Samples were boiled at 95°C for 10 min. Tissue samples were sonicated using a tip sonicator while serum samples were sonicated using water bath sonicator. Tissue lysates were then centrifuged at 16000 g for 10 min and the supernatant was collected for protein digestion. Proteins were digested using the endoproteinases LysC and trypsin (1:100 w/w) at 37°C overnight with shaking. Digested peptides were acidified using 1% Trifluoroacetic acid (TFA) and purified using the StageTips containing SDB-RPS material and eluted in 1% ammonia and 80% acetonitrile. Peptides were concentrated, dried using Speed-vac and res-suspended in buffer containing 2% acetonitrile and 0.1% TFA.

##### Peptide libraries, high pH reversed-phase fractionation

For serum, skeletal muscle, VAT and liver we used peptide libraries generated in house for quantification based on data-independent acquisition (DIA). To generate the library for the pancreatic islets, 15 μg of peptides were pooled from few samples and fractionated using high pH reversed-phase chromatography.^65^ Sixteen fractions were automatically concatenated using a rotor valve shift of 90 s. Approximately 0.3 μg of each fraction were subjected to LC-MS/MS measurements via data-dependent acquisition (DDA).

##### Mass spectrometry

Peptides were measured using LC-MS instrumentation consisting of an Easy nanoflow HPLC system (Thermo Fisher Scientific, Bremen, Germany) coupled via a nanoelectrospray ion source (Thermo Fischer Scientific, Bremen, Germany) to a Q Exactive HF-X mass spectrometer. Purified peptides were separated on a 50 cm C18 column (inner diameter 75 μm, 1.8 μm beads, Dr. Maisch GmbH, Germany). Peptides from the tissue samples were loaded onto the column with buffer A (0.5% formic acid) and eluted with a 100 min linear gradient increasing from 2-40% buffer B (80% acetonitrile, 0.5% formic acid). For the peptides from serum samples 45 min linear gradient was used. After the gradient, the column was washed with 90% buffer B and re-equilibrated with buffer A.

Mass spectra were acquired in either DDA or DIA mode. For the pilot experiment where samples from six subjects were analyzed, data for tissue samples was obtained in DDA while the serum data was acquired in DIA mode. For the main experiments, data for all samples was acquired in DIA mode. For the pancreatic islets extract, the library samples were measured in DDA mode. For the DDA method, the mass spectra were acquired with automatic switching between MS and MS/MS using a top 15 method. MS spectra were acquired in the Orbitrap analyzer with a mass range of 300–1750 m/z and 60,000 resolutions at m/z 200 with a target of 3 × 10^6^ ions and a maximum injection time of 25 ms. HCD peptide fragments acquired at 27 normalized collision energy were analyzed at 15000 resolution in the Orbitrap analyzer with a target of 1 × 105 ions and a maximum injection time of 28 ms. A DIA MS method was used for all tissue proteome measurements in which one full scan (300 to 1650 *m/z*, resolution = 60,000 at 200 *m/z*) at a target of 3 × 10^6^ ions was first performed, followed by 32 windows with a resolution of 30000 where precursor ions were fragmented with higher-energy collisional dissociation (stepped collision energy 25%, 27.5%, 30%) and analyzed with an AGC target of 3 × 10^6^ ions and maximum injection time at 54 ms in profile mode using positive polarity. For the serum samples, a DIA MS method in which one full scan (300 to 1650 *m/z*, resolution = 120,000 at 200 *m/z*) at a target of 3 x 10^6^ ions was first performed, followed by 22 windows with a resolution of 30000 where precursor ions were fragmented with higher-energy collisional dissociation (stepped collision energy 25%, 27.5%, 30%) and analyzed with an AGC target of 3 x 10^6^ ions and maximum injection time at 54 ms in profile mode using positive polarity.

### Quantification and statistical analysis

#### Data processing

Raw MS files from the experiments measured in the DDA mode (pancreatic islets library & tissue samples from pilot experiments) were processed using MaxQuant.^66^ MS/MS spectra were searched by the Andromeda^67^ search engine (integrated into MaxQuant) against the decoy UniProt-human database (downloaded in December 2017) with forward and reverse sequences.^68^ In the main Andromeda search precursor, mass and fragment mass were matched with an initial mass tolerance of 6 ppm and 20 ppm, respectively. The search included variable modifications of methionine oxidation and N-terminal acetylation and fixed modification of carbamidomethyl cysteine. The false discovery rate (FDR) was estimated for peptides and proteins individually using a target-decoy approach allowing a maximum of 1% false identifications from a revered sequence database. Raw files acquired in the DIA mode were processed using Biognosys Spectronaut software version 13.^69^ A single peptide library was generated in Spectronaut using the combined MaxQuant search results for the DDA runs from the pancreatic islets sample. The experimental DIA runs were then analyzed in Spectronaut using default settings.

#### Computational analysis

We used precompiled gmt files containing collections of GO terms (C5 release September 19, 2020) and biological pathways for KEGG and Reactome (C2 release September 19, 2020) from the molecular signatures database (MSigDB v7.2).^70^, ^71^, ^72^, ^73^, ^74^ The collection of gmt files was initially used to compile a universe of unique gene names. For each quantified protein, DIA provides an ordered list of UniProt identifiers and gene names ranked based on statistical confidence. The universe was used as means to decide on the unique gene names that most accurately represent the quantified proteins. If one or more identifiers from the ordered list intersected with the universe then the first hit from the ordered list was selected, otherwise the first, and most significant, of the three identifiers was used.

Raw data was log_2_-transformed to better approximate a normal distribution and proteins identified in at least 80% of the samples were retained for downstream analysis (Table S1E). Abundancies of proteins corresponding to the same gene in each tissue were averaged within samples. Samples with consistent large deviations from the median abundancies of all samples across tissues were excluded from the analysis (Figure S1A and Table S1E) and normalization was performed using median sweeping (Figures S1B and S1C). Missing protein levels were strongly biased for proteins located on the lower end of the detection limit (Figure S1D); hence, in order to impute the missing values, we selected a method that draws random values from a truncated distribution with parameters estimated from quantile regression (QRLIC) using the function *impute*.*QRILC* from the R package *imputeLCMD* (Figure S1E).

#### Differential analysis

The differential analysis was performed on pairwise comparisons among CTRL, PD and T2D, and the merged groups CTRL + PD and PD + T2D using the R package *limma*.^75^ The differential models were corrected for confounding factors explaining ≥1% of the median variance across proteins in at least one tissue (Table S1F).^76^ Raw p values were FDR-corrected to q-values, and they were subsequently combined with the log-fold change (logFC) to compute π-values: *π=logFC × -log*_*10*_*(q-value)*.^77^^,^^78^

#### GO and pathway enrichment analysis

Lists of proteins ranked on π-values from the differential analysis were imported into the functions *cameraPR* and *fgsea* from the R packages *limma* and *fgsea*, respectively. *cameraPR* was executed on default settings, while *fgsea* was set to perform 1,000,000 permutations, and to consider only terms with ≥5 and ≤300 proteins. For both methods the enrichment of GO terms and biological pathways was performed separately using the *gmt* files described above and the raw p-values were corrected for multiple testing using the R package *qvalue*. As the collection of enriched GO terms and pathways the intersection of the significant terms (q < 0.05) sharing the same direction of change in both methods was considered.

Enrichment analysis of biological terms for pre-defined sets of proteins identified only in one tissue (tissue-specific) and shared among tissues (tissue-shared) was performed using a hypergeometric test. The background set for tissue-shared proteins was the union of proteins identified across tissues, while as background set for tissue-specific proteins was used the collection of proteins in the respective tissue. Sets with ≥5 and ≤300 proteins were retained, and p-values were corrected for multiple testing using the R package *qvalue*.

## Data Availability

The mass spectrometry proteomics data have been deposited to the ProteomeXchange Consortium (http://proteomecentral.proteomexchange.org) via the PRIDE partner repository under the accession number PRIDE: PXD027597.^62^ This data will be publicly available at the date of publication. Accession numbers are listed in the key resources table. Clinical data is provided in supplementary tables. The pipeline for the analysis is available on GitHub *https://github.com/klevdiamanti/multitissue_ms_proteomics*. A web-based tool that allows visualization and exploration for levels of proteins across tissues is available on *http://bioinf.icm.uu.se:3838/multitissue_ms_proteomics/*. Any additional information required to reanalyze the data reported in this paper is available from the lead contact upon request.

## Supplemental information

Document S1. Figures S1–S13

Table S1. Details of the characteristics of the clinical, anthropometric, and technical variables

Document S2. Article plus supplemental information
