# Supplementary material for: Organ-specific metabolic pathways distinguish prediabetes, type 2 diabetes, and normal tissues
Source: Cell Rep Med. 2022 Oct 4;3(10):100763. doi: 10.1016/j.xcrm.2022.100763 (PMC9589007; doi:10.1016/j.xcrm.2022.100763)
Supplement: Document S1. Figures S1–S13 [file mmc1.pdf]

Cell Reports Medicine, Volume *3*

Supplemental information

Organ-specific metabolic pathways distinguish prediabetes, type 2 diabetes, and normal tissues

Klev Diamanti, Marco Cavalli, Maria J. Pereira, Gang Pan, Casimiro Castillejo-López, Chanchal Kumar, Filip Mundt, Jan Komorowski, Atul S. Deshmukh, Matthias Mann, Olle Korsgren, Jan W. Eriksson, and Claes Wadelius

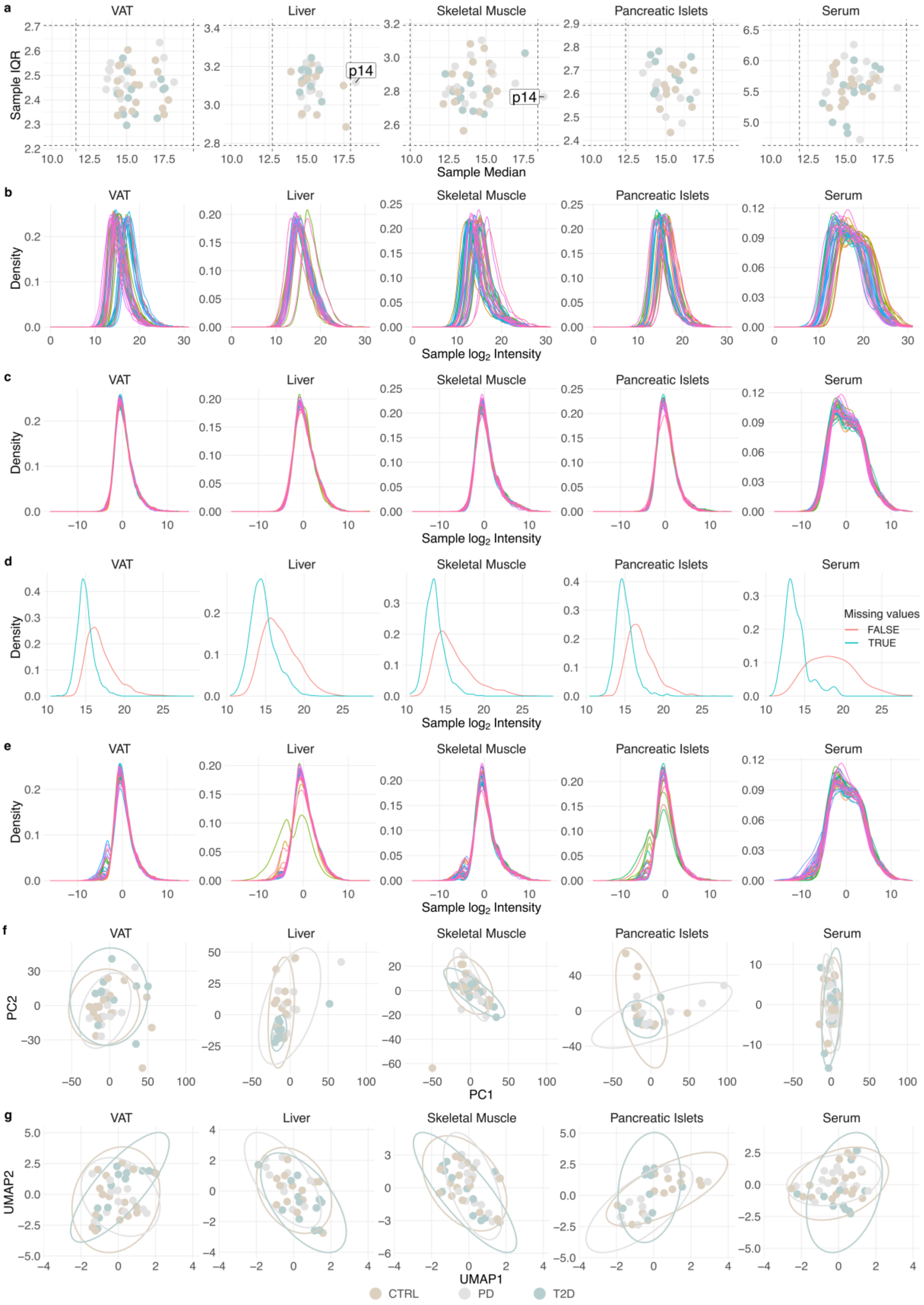

Figure S1: Quality control of MS proteomics data in each tissue. a) Median expression level of all proteins of each sample (x axis) against the interquartile range (IQR) of the expressed proteins in each sample (y axis). The dotted frame indicates ±3 standard deviations (SDs) from the average median of all samples and the average IQR of all samples, respectively. Labelled samples deviate more than 3 SDs from the averages. **b)** Distribution of raw

log_2-transformed protein levels. Each line represents one sample. **c)** Distribution of median-shifted normalized protein levels. **d)** Distribution of the abundance of proteins with and without missing values. **e)** Distribution of protein abundancies after QRILC lower-tail imputation. **f)** The two first principal components (PCs) from the principal component analysis (PCA) that was performed on the normalized imputed dataset using the *pca* function from the R package *pcaMethods* with the selected method being "ppca". **g)** The two first components from the uniform manifold approximation and projection (UMAP) that was performed on the normalized imputed dataset. The number of neighbors for UMAP was set to five.

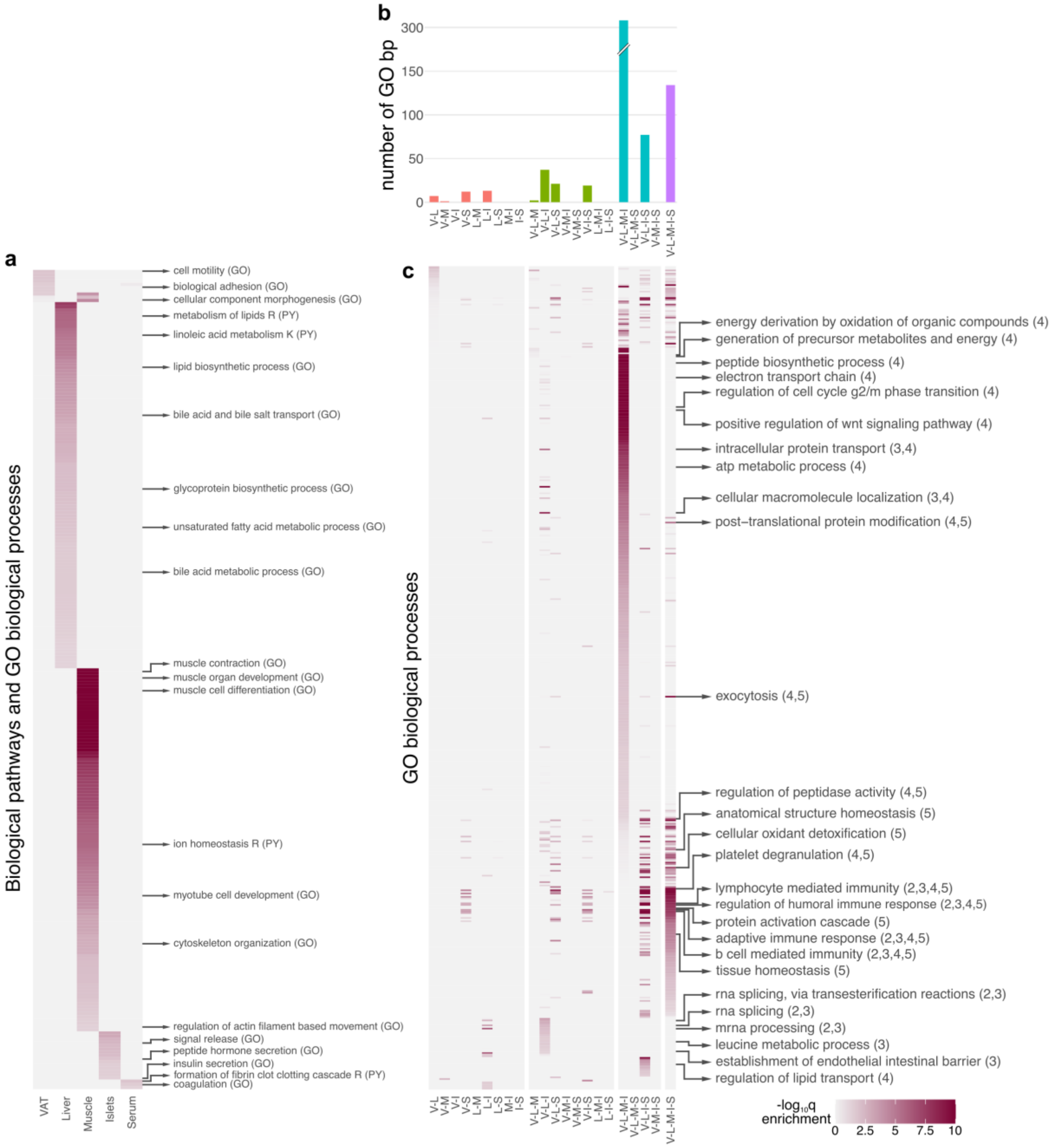

Figure S2: Exploration of tissue-specific and tissue-shared proteins. a) Significantly enriched (q<0.05) GO terms for biological processes and biological pathways for proteins identified only in one tissue (tissue-specific). Columns represent tissues and rows represent enriched terms. Values in the parentheses of the terms mark the origin of the term: GO for gene ontology (GO) terms and PY for pathways. Terms representing pathways contain R or K as suffix to signify Reactome or KEGG pathways, respectively. A detailed list of the enriched terms is shown in (Table S1j). **b-c)** GO terms for biological processes for proteins shared among tissues (tissue-shared). **b)** Shows the number of enriched terms for each combination of tissues. **c)** Panels from left to right represent proteins shared exclusively among two, three, four and five tissues. Columns represent tissue combinations and rows represent enriched GO terms. Column names are abbreviations of the tissue names: VAT (V), liver (L), skeletal muscle (M), pancreatic islets (I) and serum (S). Values in the parentheses of the GO terms mark the panel(s) in which the term is enriched. 2 implies combinations of 2 tissues, 3 combination of 3 tissues and so on. A detailed list of the enriched terms is shown in (Table S1k).

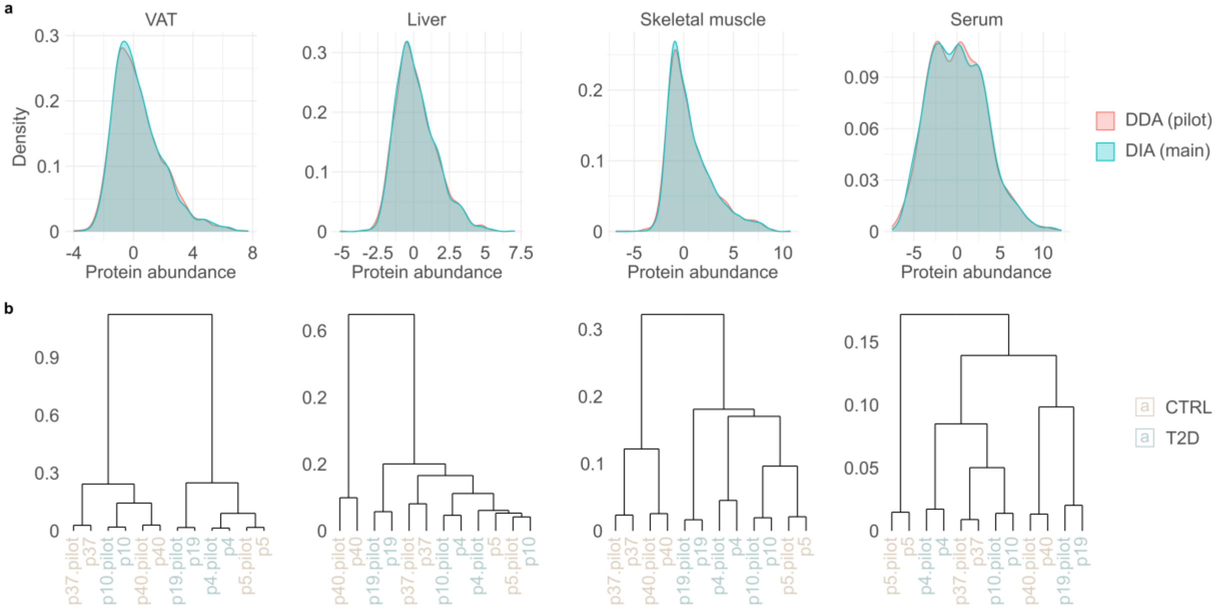

Figure S3: Comparison and clustering of six samples that were analyzed in both pilot and main analyses. Pancreatic islets were excluded from the analysis due to low quality of four samples, which were consequently excluded from the main analysis. Samples included in this analysis are equally distributed between CTRL and T2D. Pilot study is marked as data-dependent acquisition (DDA) and the main one as data-independent acquisition (DIA). The data was $\log_2$-transformed and the set of shared top-500 most abundant proteins was analyzed. Batch effect was corrected using the function *removeBatchEffect* from the R package *limma*.
a) Distribution of proteins in the pilot and main run after batch effect correction. **b)** Hierarchical clustering (HC) of DDA and DIA samples. DDA samples were marked with the suffix “pilot”. *1-r*, where *r* is Pearson correlation coefficient, was used as the distance metric and the *ward.D* algorithm was used for the HC.

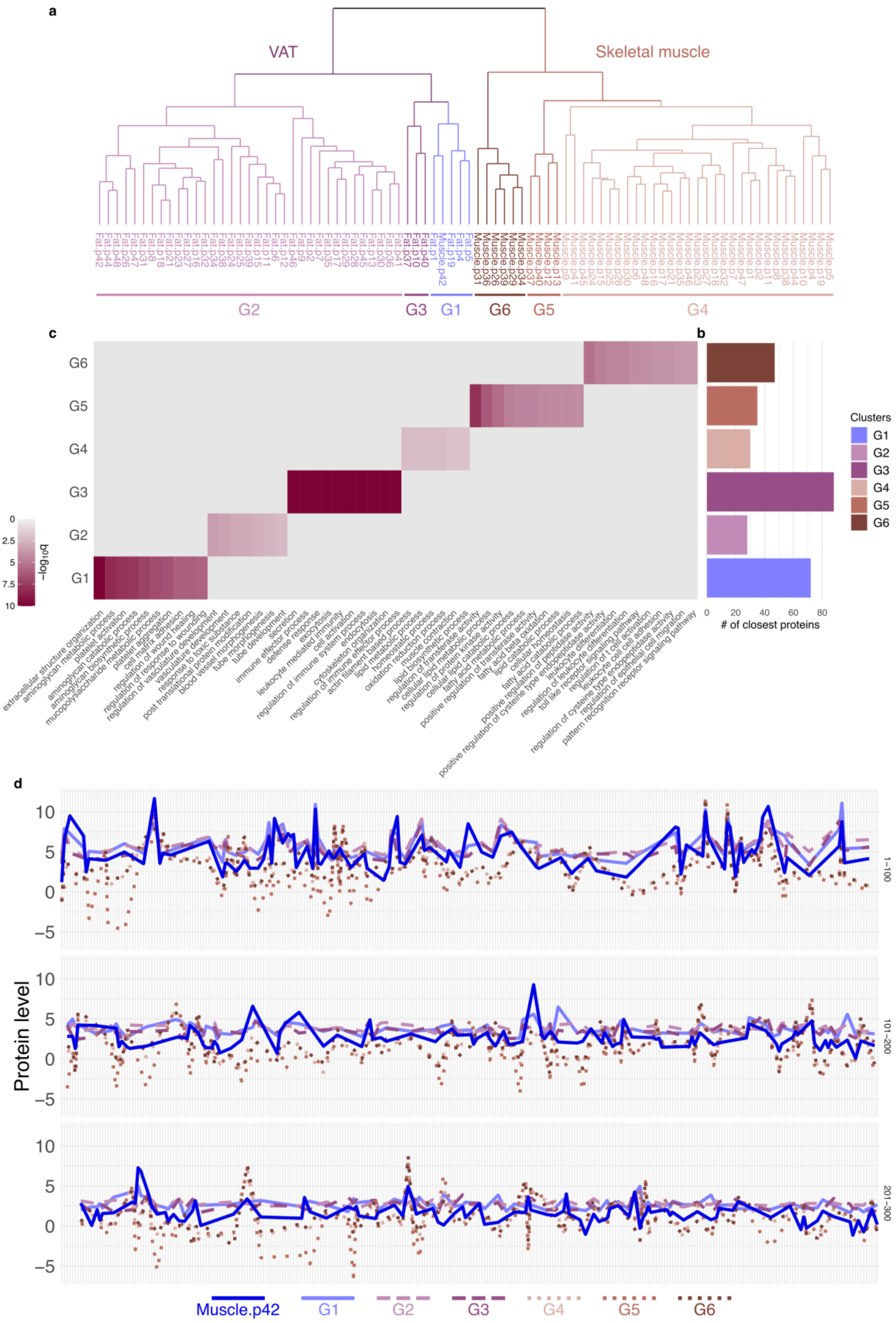
a
VAT
Skeletal muscle
G2
G3
G1
G6
G5
G4
c
b
Clusters
G1
G2
G3
G4
G5
G6
# of closest proteins
−log10q
d
Protein level
1–100
101–200
201–300
Muscle.p42

Figure S4: Investigation of the misclassified sample p42 from skeletal muscle. a) Zoom into the hierarchical clustering (HC) from (Figure 1c) for VAT and skeletal muscle. **b)** Number of the top 300 most abundant proteins shared between VAT and skeletal muscle that are closer to sample p42 from skeletal muscle. Proteins for each group defined from HC have been collapsed to the average of their respective groups. **c)** Top 10 most significantly enriched GO terms from the GO enrichment analysis performed on the lists of proteins of each group in *b*. **d)** Expression of the top 300 most abundant proteins shared between VAT and skeletal muscle. p42 in skeletal muscle is shown independently while proteins from the other groups were collapsed to the average of the respective group.

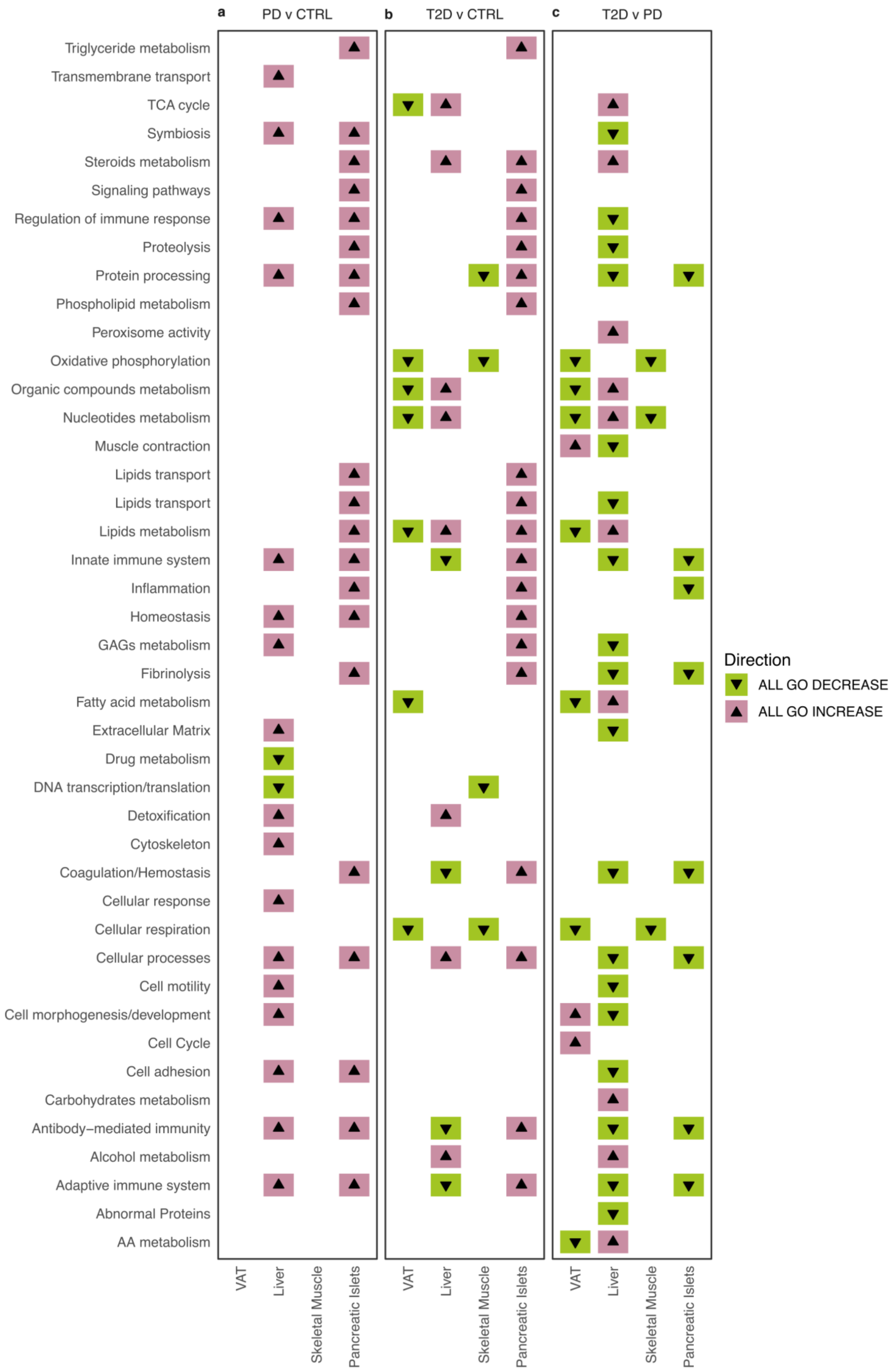

a PD v CTRL
b T2D v CTRL
c T2D v PD
Triglyceride metabolism
Transmembrane transport
TCA cycle
Symbiosis
Steroids metabolism
Signaling pathways
Regulation of immune response
Proteolysis
Protein processing
Phospholipid metabolism
Peroxisome activity
Oxidative phosphorylation
Organic compounds metabolism
Nucleotides metabolism
Muscle contraction
Lipids transport
Lipids transport
Lipids metabolism
Innate immune system
Inflammation
Homeostasis
GAGs metabolism
Fibrinolysis
Fatty acid metabolism
Extracellular Matrix
Drug metabolism
DNA transcription/translation
Detoxification
Cytoskeleton
Coagulation/Hemostasis
Cellular response
Cellular respiration
Cellular processes
Cell motility
Cell morphogenesis/development
Cell Cycle
Cell adhesion
Carbohydrates metabolism
Antibody–mediated immunity
Alcohol metabolism
Adaptive immune system
Abnormal Proteins
AA metabolism
VAT
Liver
Skeletal Muscle
Pancreatic Islets
Direction
ALL GO DECREASE
ALL GO INCREASE

Figure S5: Direction of change for curated groups of GO terms for significantly enriched biological processes ($q<0.01$). a-c) Panels represent pair-wise comparisons of phenotypes, columns represent tissues and rows represent curated groups of GO terms for biological processes. A combination of a triangle pointing upwards and a pink background color implies increase in the group, while those pointing downwards in combination with green background imply decrease. A graphic representation of the individual GO terms and as well as the curated groups is shown in (Figure 2), and detailed information on grouping and significance is shown in (Table S1m).

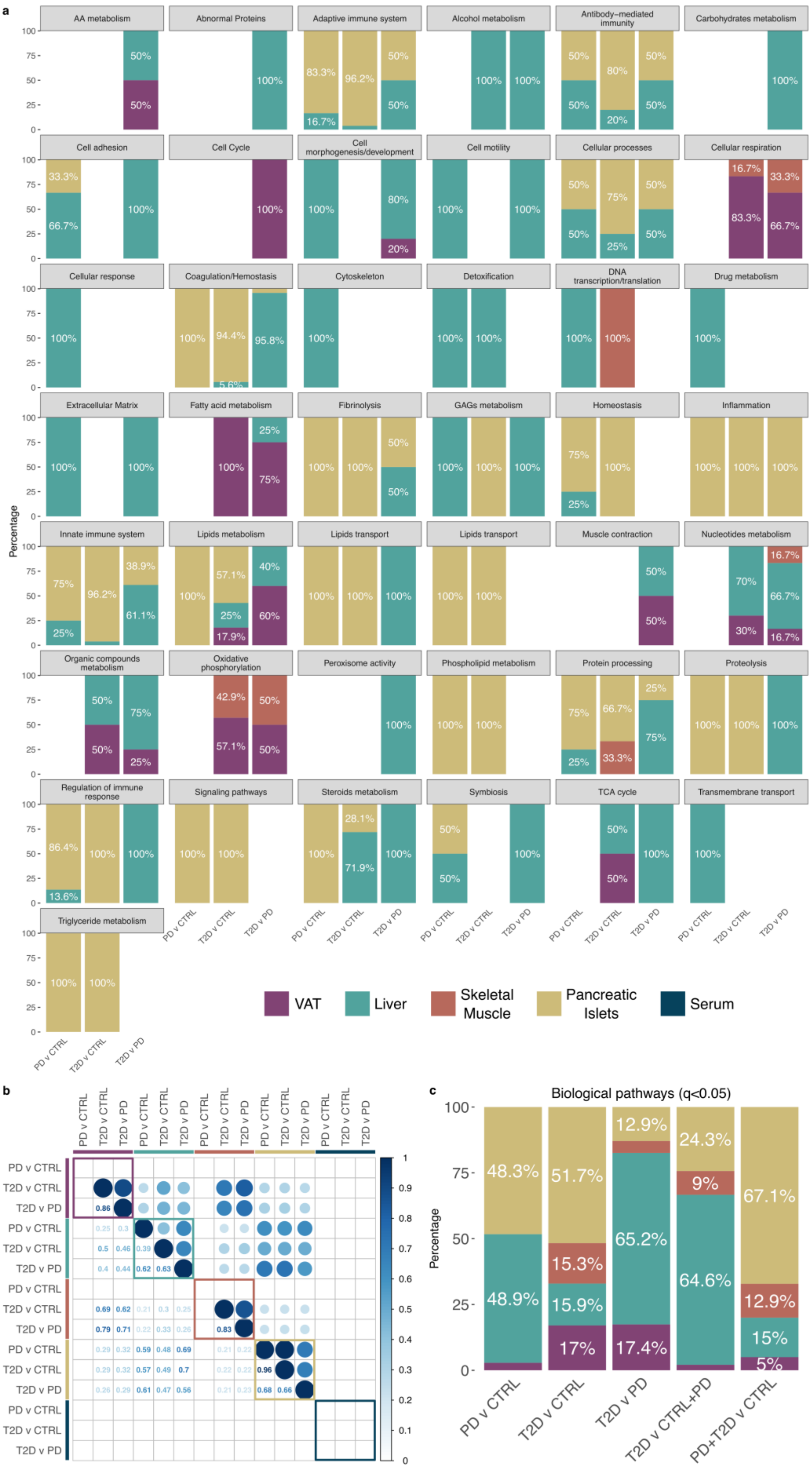
a
AA metabolism
Abnormal Proteins
Adaptive immune system
Alcohol metabolism
Antibody–mediated immunity
Carbohydrates metabolism
Cell adhesion
Cell Cycle
Cell morphogenesis/development
Cell motility
Cellular processes
Cellular respiration
Cellular response
Coagulation/Hemostasis
Cytoskeleton
Detoxification
DNA transcription/translation
Drug metabolism
Extracellular Matrix
Fatty acid metabolism
Fibrinolysis
GAGs metabolism
Homeostasis
Inflammation
Innate immune system
Lipids metabolism
Lipids transport
Lipids transport
Muscle contraction
Nucleotides metabolism
Organic compounds metabolism
Oxidative phosphorylation
Peroxisome activity
Phospholipid metabolism
Protein processing
Proteolysis
Regulation of immune response
Signaling pathways
Steroids metabolism
Symbiosis
TCA cycle
Transmembrane transport
Triglyceride metabolism
Percentage
PD v CTRL
T2D v CTRL
T2D v PD
VAT
Liver
Skeletal Muscle
Pancreatic Islets
Serum
b
c
Biological pathways (q<0.05)
Percentage
PD v CTRL
T2D v CTRL
T2D v PD
T2D v CTRL+PD
PD+T2D v CTRL

Figure S6: Distributions and similarities of enriched GO biological processes and biological pathways across tissues for pairwise comparisons of CTRL, PD, T2D, and their merged groups CTRL+PD and PD+T2D. a) Distribution of enriched GO biological processes (q<0.01) across curated groupings and pair-wise phenotype comparisons among CTRL, PD and T2D. For groups of GO biological processes from (Figure 2) the fraction of terms from tissues of origin was calculated. Details on the groups are shown in (Table S1m).
b) Similarity of GO biological terms for pair-wise comparisons of CTRL, PD and T2D across tissues**.** The similarity analysis was performed on sets of enriched GO biological processes (q<0.01) using the function *mgoSim* from the R package *GOSemSim* to obtain a combined similarity score with parameters *measure="Wang"* and *combine="BMA"*. **c)** Distribution of enriched biological pathways at 5% FDR corresponding to (Figure 3 and Table S1n).

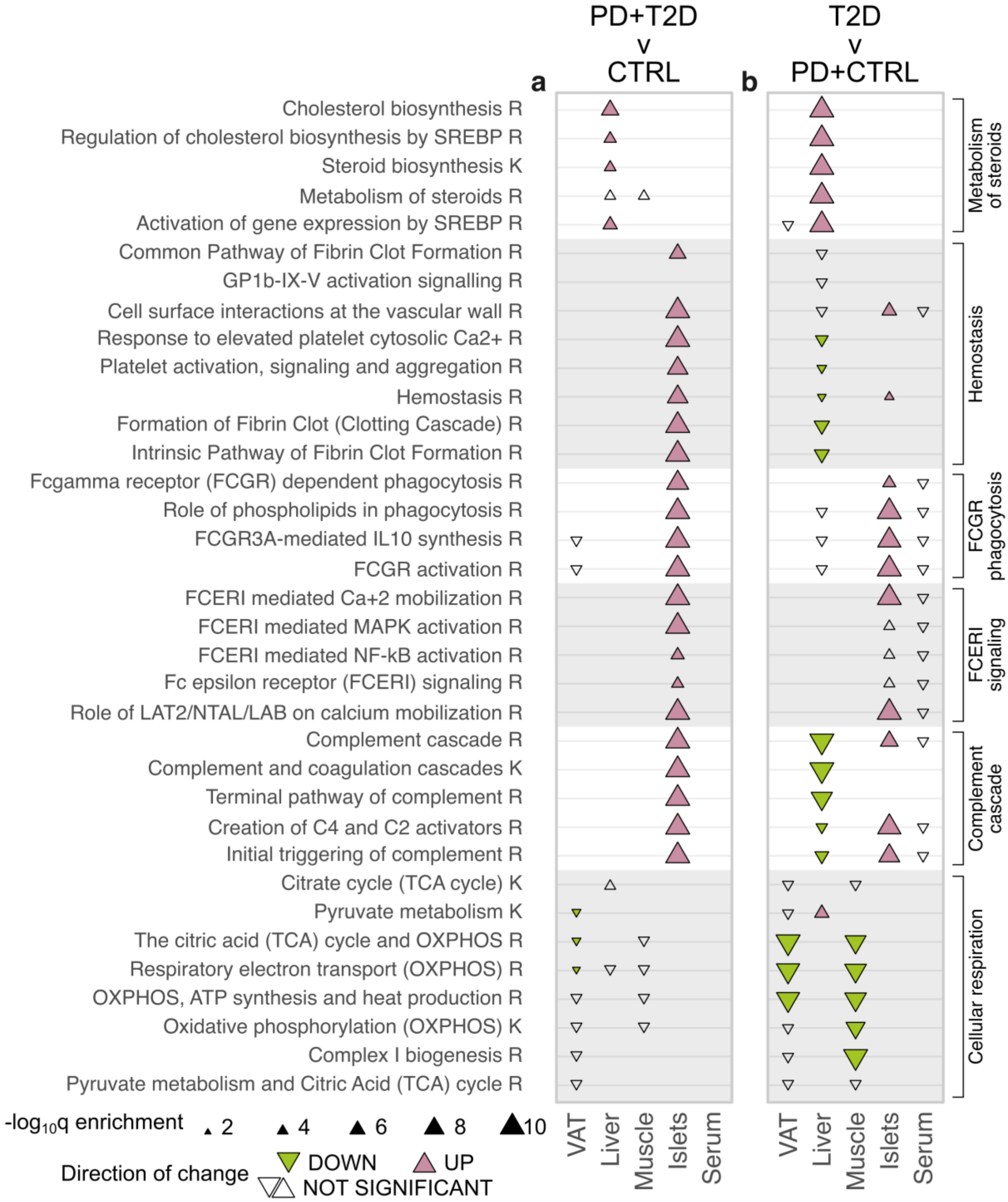

Figure S7: Selected groups of enriched biological pathways (q≤0.05) for comparisons of the merged groups of CTRL+PD and PD+T2D to T2D and CTRL, respectively. The plot contains an identical set to the one shown in (Figure 3). Colored triangles indicate statistical significance (q<0.05) and direction. Rows represent pathways, panels represent pair-wise comparisons and columns within panels represent tissues. The database of origin for each pathway is noted with a suffix R or K implying Reactome or KEGG, respectively. A full list of enriched pathways is shown in (Table S1n). Sterol regulatory element binding proteins have been abbreviated as SREBP and Glycoprotein Ib-IX-V has been abbreviated as GP1b-IX-V.

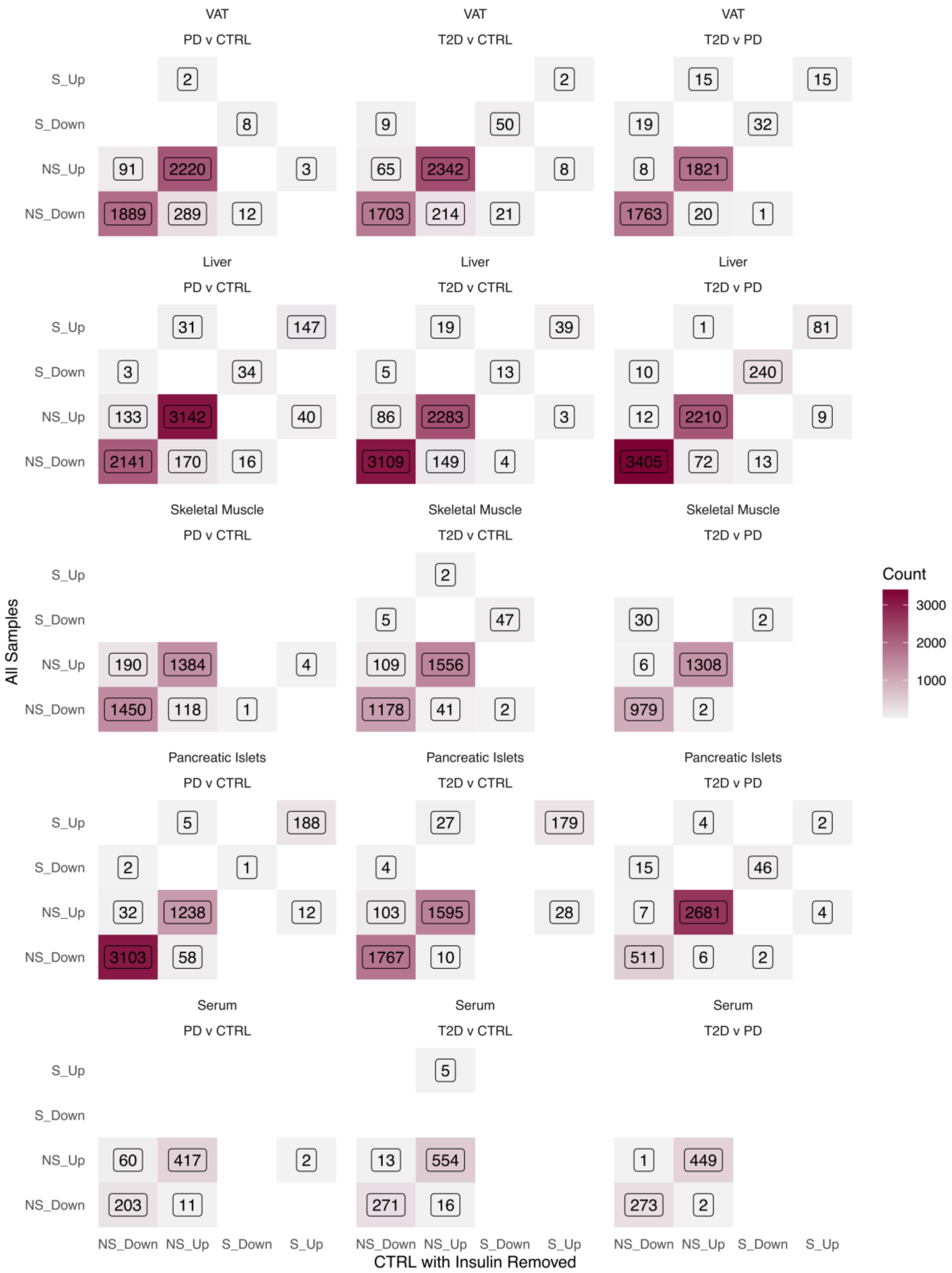

Figure S8: Aggregated count of significance and enrichment direction of GO terms and biological pathways before and after exclusion of the three subjects from CTRL that were administered insulin while in ICU. Each tile represents count of significance and direction of enrichment of biological pathways and GO terms between the original and the recomputed set of results after exclusion of the three subjects from CTRL that were administered insulin while in ICU. X-axis represents GO terms and pathways from the original analysis, and y-axis represents GO terms and pathways from the analysis excluding these three subjects. Labels

in both axes are named after A_B, where “A” represents significance (S) or Non-Significance (NS), and “B” represents enrichment direction Up or Down.

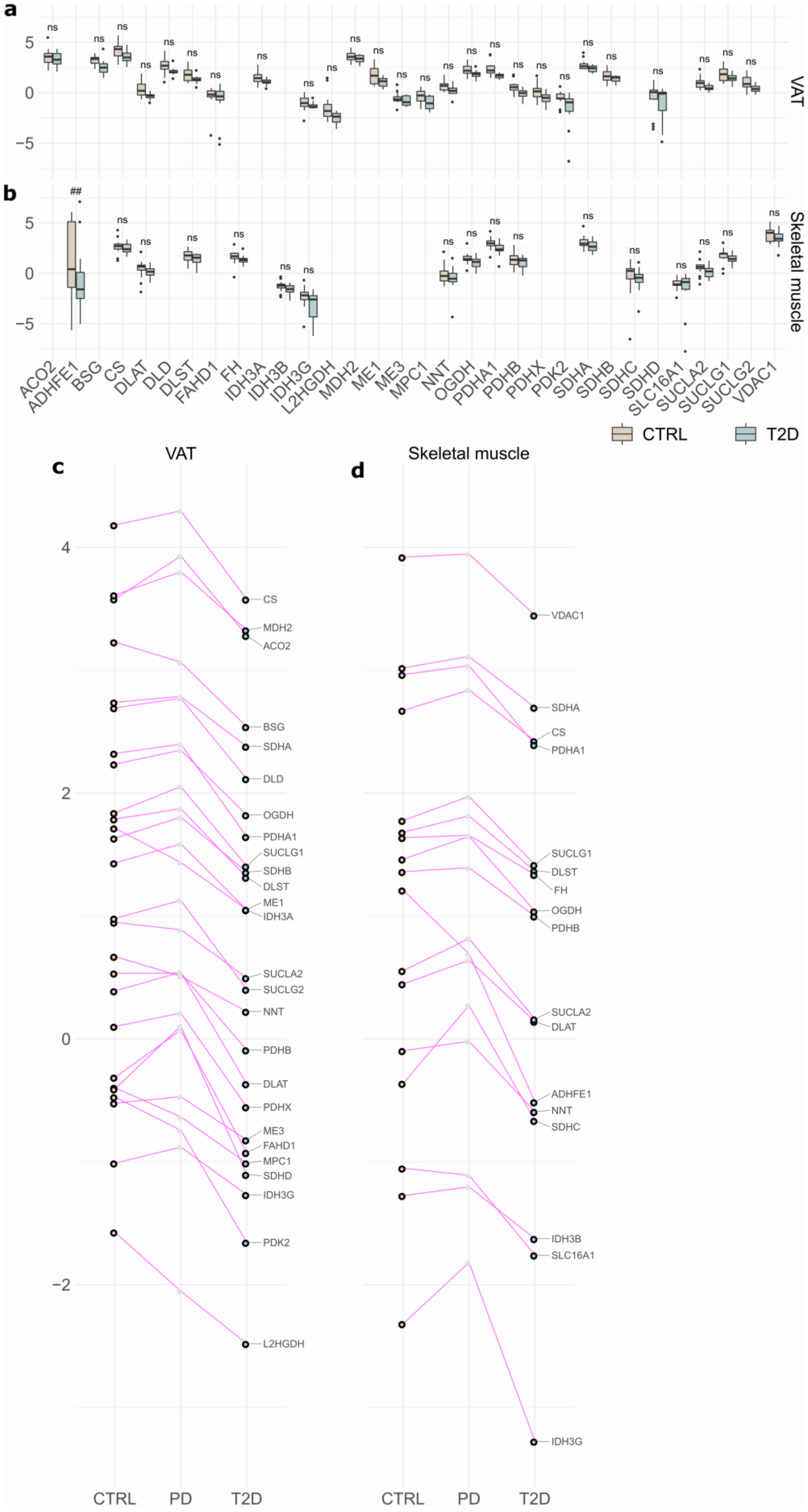

a
b
VAT
Skeletal muscle
ACO2
ADHFE1
BSG
CS
DLAT
DLD
DLST
FAHD1
FH
IDH3A
IDH3B
IDH3G
L2HGDH
MDH2
ME1
ME3
MPC1
NNT
OGDH
PDHA1
PDHB
PDHX
PDK2
SDHA
SDHB
SDHC
SDHD
SLC16A1
SUCLA2
SUCLG1
SUCLG2
VDAC1
CTRL
T2D
c
VAT
d
Skeletal muscle
CTRL
PD
T2D

Figure S9: Details on levels of proteins for TCA cycle and pyruvate metabolism in VAT and skeletal muscle for T2D and CTRL. a-b) The two boxplots display differences in expression levels in **a)** VAT and **b)** skeletal muscle samples between CTRL and T2D. ***: $\pi \leq 2.1281$, **: $\pi \leq 1.1733$, *: $\pi \leq 0.6274$, #: $\pi \leq 0.4292$, ##: $\pi \leq 0.2572$, ns: $\pi > 0.2572$. **c-d)** The two bottom plots illustrate the overall trend of changes in expression of proteins characterized as driving for the TCA cycle and pyruvate metabolism pathway across CTRL, PD and T2D in **c)** VAT and **d)** skeletal muscle. Dots show average protein expression and a black circle around dots marks the outcomes that are being compared in the current example. Lines are used to mark alterations across phenotypes for a given protein.

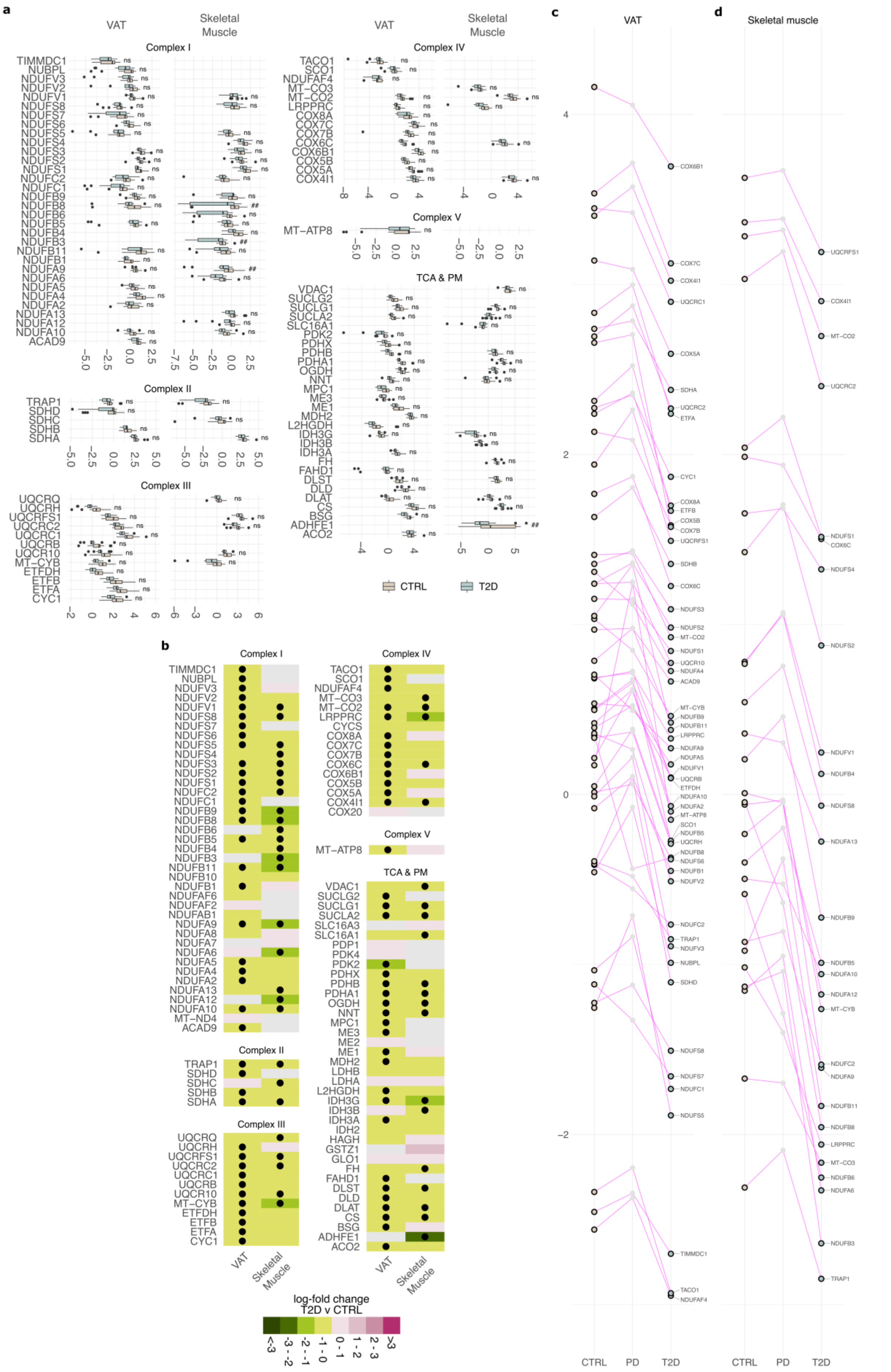
a
VAT
Skeletal Muscle
Complex I
Complex II
Complex III
Complex IV
Complex V
TCA & PM
CTRL
T2D
b
Complex I
Complex II
Complex III
Complex IV
Complex V
TCA & PM
VAT
Skeletal Muscle
log-fold change
T2D v CTRL
<-3
-3 - -2
-2 - -1
-1 - 0
0 - 1
1 - 2
2 - 3
>3
c
VAT
d
Skeletal muscle
CTRL
PD
T2D

Figure S10: Details on levels of proteins for oxidative phosphorylation, ATP synthesis by chemiosmotic coupling, and heat production by uncoupling proteins in VAT and skeletal muscle for T2D and CTRL. **a-b)** Boxplot and heatmap were stratified based on mitochondrial protein complexes. Mitochondrial complexes were obtained from the HUGO database (group 639) release January 31, 2021. **a)** Boxplots display differences in expression levels in VAT and skeletal muscle samples between CTRL and T2D. ***: $\pi \leq 2.1281$, **: $\pi \leq 1.1733$, *: $\pi \leq 0.6274$, #: $\pi \leq 0.4292$, ##: $\pi \leq 0.2572$, ns: $\pi > 0.2572$. **b)** Heatmap shows log-fold change of proteins identified in VAT and skeletal muscle. Black dots mark driving proteins in altering the pathway. Grey tiles mark non identified proteins in the respective tissue. **c-d)** Overall trend of changes in expression of proteins characterized as driving for the oxidative phosphorylation, ATP synthesis by chemiosmotic coupling, and heat production by uncoupling proteins pathway across CTRL, PD and T2D for **c)** VAT and **d)** skeletal muscle. Dots show average protein expression and a black circle around dots marks the outcomes that are being compared in the current example. Lines are used to mark alterations across phenotypes for a given protein.

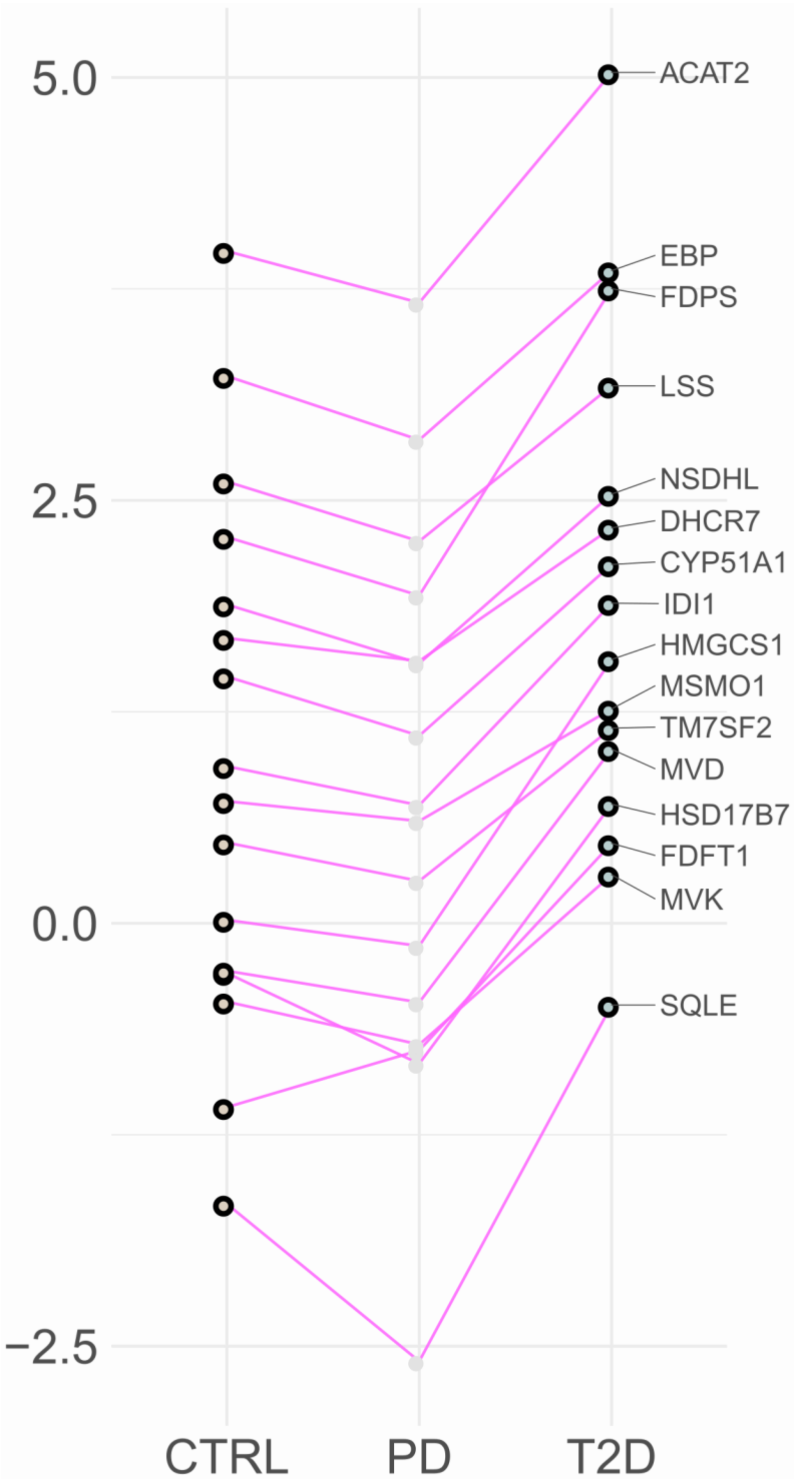

Figure S11: Overall trend of changes in expression of proteins characterized as driving for the cholesterol biosynthesis pathway in liver across CTRL, PD and T2D. Dots show average protein expression and a black circle around dots marks the outcomes that are being compared in the current example. Lines are used to mark alterations across phenotypes for a given protein.

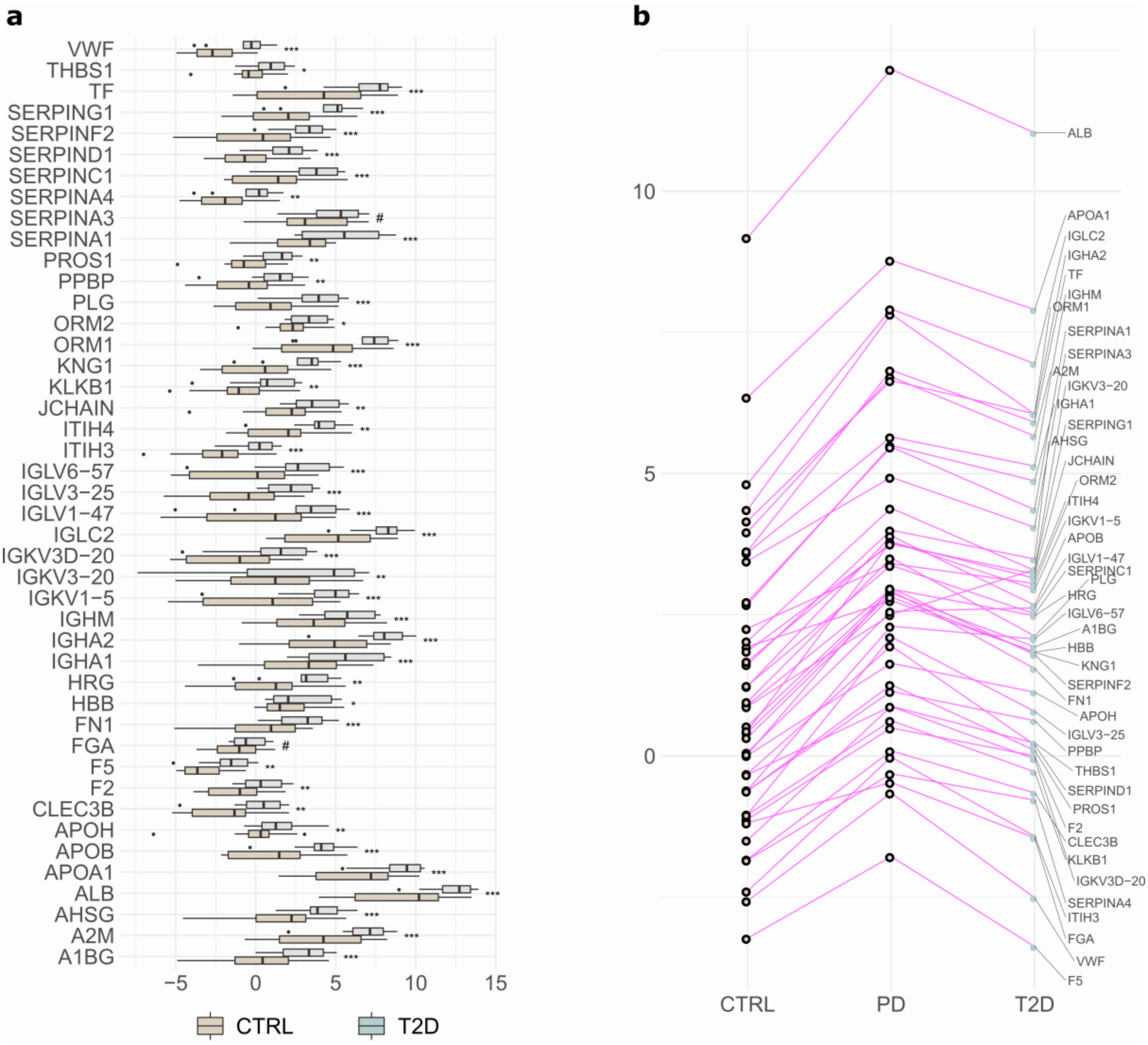

Figure S12: Details on levels of proteins for hemostasis in pancreatic islets for PD and CTRL.
a) Differences in expression levels between CTRL and PD. ***: π≤2.1281, **: π≤1.1733, *: π≤0.6274, #: π≤0.4292, ##: π≤0.2572, ns: π>0.2572. **b)** Overall trend of changes in expression of proteins characterized as driving for the hemostasis pathway in pancreatic islets across CTRL, PD and T2D. Dots show average protein expression and a black circle around dots marks the outcomes that are being compared in the current example. Lines are used to mark alterations across phenotypes for a given protein.

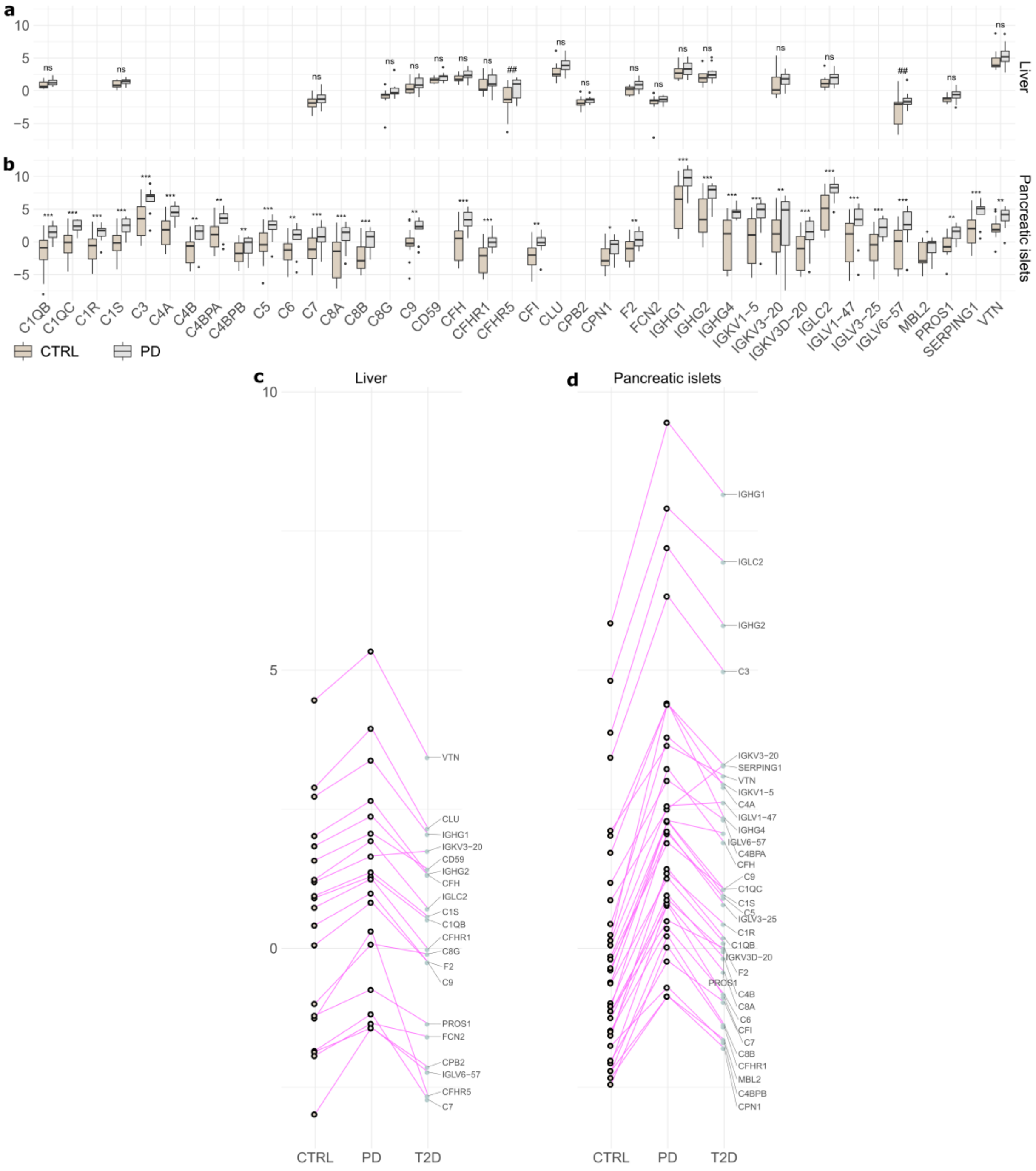

Figure S13: Details on levels of proteins for complement cascade in liver and pancreatic islets for PD and CTRL. a-b) The boxplots display differences in expression levels from **a)** liver and **b)** pancreatic islets samples between CTRL and PD. ***: $\pi \leq 2.1281$, **: $\pi \leq 1.1733$, *: $\pi \leq 0.6274$, #: $\pi \leq 0.4292$, ##: $\pi \leq 0.2572$, ns: $\pi > 0.2572$. **c-d**) Overall trend of changes in expression of proteins characterized as driving for the complement cascade pathway across CTRL, PD and T2D in **c)** liver and **d)** pancreatic islets. Dots show average protein expression and a black circle around dots marks the outcomes that are being compared in the current example. Lines are used to mark alterations across phenotypes for a given protein.
